# Supplementary material for: Experiences along the diagnostic pathway for patients with advanced lung cancer in the USA: a qualitative study
Source: BMJ Open. 2021 Apr 22;11(4):e045056. doi: 10.1136/bmjopen-2020-045056 (PMC8070881; doi:10.1136/bmjopen-2020-045056)
Supplement: Supplementary data [file bmjopen-2020-045056supp001.pdf]

Intial question.

Share with me the whole story of the cancer diagnosis the way that you would tell it to a friend. Tell me every detail of the whole story of the cancer diagnosis from the very first time when you noticed that something is not right.

Follow up prompts

1. Can you elaborate on some of these specific turning points to reflect on some of the conversations that took place?
2. Besides the [first symptom], what else have you had in the period before the diagnosis of cancer?
3. Tell me more about the conversations with the doctor the first time you had symptoms.
4. Can you share about when your doctor started to be alarmed?
5. Then you had the visit to the [Urgent Care, PCP office] where they did an x-ray, and they found [tumor, fluids, etc.], walk me through the process.
6. Tell me how the results came and how they were conveyed to you. What happened after?
7. They found a tumor. What was its size, and where was it located?
8. How did she share the findings on the [diagnostic test] with you?
9. Please share with me some of your earlier reactions when your primary care doctor gave you the results.
10. How was the process of getting a CT scan?
11. Please walk me through your first interaction with the specialist, the team, or the doctor who was not your primary care.
12. They did a biopsy after that. Please walk me through some of the procedures, the decision around that.
13. Who gave you the cancer diagnosis, and can you walk me through the conversation that took place the first time they confirmed the diagnosis?
14. When the conversation came around the metastatic disease, what were some of your thoughts and feelings at that time?
15. How was your experience with the oncologist?
16. Can you share your conversations with the oncologist?
17. Do you mind sharing what was going on in your mind after the first interaction with the oncologist?
18. When were you told it was [ALK, EGFR, ROS1].
19. Did anybody explain to you what it meant back?
20. Can you explain what [ALK, EGFR, ROS1] means? Assume that I don't know anything about that.
21. Then a few days the results of the [ALK, EGFR, ROS1] came back positive. Tell me how you received the news about [ALK, EGFR, ROS1].
22. Did they offer a management plan, treatment plan early on?
23. How were some of those decisions made to start chemotherapy and radiation?
24. What were conversations around starting the [targeted therapy]?
