# Supplementary material for: Experiences along the diagnostic pathway for patients with advanced lung cancer in the USA: a qualitative study
Source: BMJ Open. 2021 Apr 22;11(4):e045056. doi: 10.1136/bmjopen-2020-045056 (PMC8070881; doi:10.1136/bmjopen-2020-045056)
Supplement: Author's manuscript [file bmjopen-2020-045056.draft_revisions.pdf]

BMJ Open is committed to open peer review. As part of this commitment we make the peer review history of every article we publish publicly available.

When an article is published we post the peer reviewers' comments and the authors' responses online. We also post the versions of the paper that were used during peer review. These are the versions that the peer review comments apply to.

The versions of the paper that follow are the versions that were submitted during the peer review process. They are not the versions of record or the final published versions. They should not be cited or distributed as the published version of this manuscript.

BMJ Open is an open access journal and the full, final, typeset and author-corrected version of record of the manuscript is available on our site with no access controls, subscription charges or pay-per-view fees (http://bmjopen.bmj.com).

If you have any questions on BMJ Open's open peer review process please email info.bmjopen@bmj.com

BMJ Open

Experiences along the diagnostic pathway for patients with advanced lung cancer

Journal:	*BMJ Open*
Manuscript ID	bmjopen-2020-045056
Article Type:	Original research
Date Submitted by the Author:	21-Sep-2020
Complete List of Authors:	Al Achkar, Morhaf; University of Washington, Family Medicine ;<br>Zigman Suchsland, Monica; University of Washington,<br>Walter, Fiona; University of Cambridge, Dept of Public Health and Primary Care<br>Neal, Richard; University of Leeds,<br>Goulart, BH; University of Washington,<br>Thompson, Matthew; University of Washington, Department of Family Medicine
Keywords:	Respiratory tract tumours < ONCOLOGY, QUALITATIVE RESEARCH, Cancer genetics < GENETICS

SCHOLARONE™
Manuscripts

I, the Submitting Author has the right to grant and does grant on behalf of all authors of the Work (as defined in the below author licence), an exclusive licence and/or a non-exclusive licence for contributions from authors who are: i) UK Crown employees; ii) where BMJ has agreed a CC-BY licence shall apply, and/or iii) in accordance with the terms applicable for US Federal Government officers or employees acting as part of their official duties; on a worldwide, perpetual, irrevocable, royalty-free basis to BMJ Publishing Group Ltd ("BMJ") its licensees and where the relevant Journal is co-owned by BMJ to the co-owners of the Journal, to publish the Work in this journal and any other BMJ products and to exploit all rights, as set out in our licence.

The Submitting Author accepts and understands that any supply made under these terms is made by BMJ to the Submitting Author unless you are acting as an employee on behalf of your employer or a postgraduate student of an affiliated institution which is paying any applicable article publishing charge ("APC") for Open Access articles. Where the Submitting Author wishes to make the Work available on an Open Access basis (and intends to pay the relevant APC), the terms of reuse of such Open Access shall be governed by a Creative Commons licence – details of these licences and which Creative Commons licence will apply to this Work are set out in our licence referred to above.

Other than as permitted in any relevant BMJ Author's Self Archiving Policies, I confirm this Work has not been accepted for publication elsewhere, is not being considered for publication elsewhere and does not duplicate material already published. I confirm all authors consent to publication of this Work and authorise the granting of this licence.

For peer review only

Experiences along the diagnostic pathway for patients with advanced lung cancer

Morhaf Al Achkar, MD, PhD

(Corresponding Author)

University of Washington

alachkar@uw.edu

Phone: (206) 520-2405

FAX: (206) 520-2450

331 NE Thornton Place

Seattle, WA, 98125, USA

Monica L Zigman Suchsland, MPH

University of Washington

Fiona M Walter, MA, MD, FRCGP

The Primary Care Unit, Department of Public Health & Primary Care, University of Cambridge, UK

Richard D Neal, PhD FRCGP

University of Leeds

Bernardo H.L. Goulart, MD, MS

University of Washington

Fred Hutchinson Cancer Research Center

Matthew Thompson, MBChB, MPH, DPhil

University of Washington

For peer review only

Word Count: 2913

Number of:

Tables: 4

Figures: 2

Appendix: 1

Keywords: lung cancer, cancer diagnosis, oncognetic alterations.

List of Abbreviations:

CT	Computed Tomography
ROS1	c-Ros oncogene 1
ALK	Anaplastic lymphoma kinase
EGFR	Epidermal growth factor receptor
NSCLC	Non-small cell lung cancer
PCP	Primary care provider
CXR	Chest X-ray
ER	Emergency room
PET	positron emission tomography

For peer review only

Abstract (248 words)

Background: Most lung cancer patients are diagnosed at advanced stages. But the advent of oral targeted therapies improved prognosis of many lung cancer patients.

Purpose: We aimed to understand the diagnostic experiences of patients with advanced lung cancer with oncogenic mutations.

Methods: Qualitative interviews with patients with advanced or metastatic non-small cell lung cancer with oncogenic alterations. Patients were recruited from online support groups within the US. Interviews were conducted remotely or in person. Analysis used an iterative inductive and deductive process. Themes were mapped to the Model of Pathways to Treatment..

Results: 40 patients (12 male and 28 female) with a median age of 48.We identified 9 distinct themes. During the "patient interval," individuals became concerned about symptoms, but often attributed them to other causes. Prolonged or more severe symptoms prompted care seeking. During the "primary care interval," doctors initially treated for illnesses other than cancer. Discovery of an imaging abnormality was a turning point in diagnostic pathwaies. Occasionally, severity of symptoms prompted patients to seek emergency care. During the "secondary care interval," obtaining tissue samples was pivotal in confirming diagnosis. Delays in accessing oncology care sometimes led to patient distress. Obtaining genetic testing was crucial in directing patients to receive targeted treatments.

Conclusions: Patients experienced multiple different routes to their diagnosis. Some patients perceived delays, inefficiencies, and lack of coordination which could be distressing. Shifting the stage of diagnosis of lung cancer to optimize the impact of targeted therapies will require concerted efforts in early detection.

For peer review only

Strengths and limitations of this study

The study's strengths include exploring the perspectives on the diagnosis journey of a large number of participants representing a relatively new group of lung cancer survivors: those on targeted therapies that experience significantly superior outcomes.

Our findings were developed within an existing theoretical framework used in research on early cancer diagnosis by many other countries.

The study's limitations include relying on individuals identified from lung cancer survivor groups, which may have reduced the representativeness, particularly of individuals from less affluent backgrounds.

Only a small proportion of our participants experienced barriers in accessing care due to financial concerns, which may have limited our ability to determine these factors' impact.

Recall bias and differential recall bias are major concerns with this type of research.

For peer review only

Background

Lung cancer is the leading cause of cancer death and the second most common cancer type in the United States (US).[1] In 2016, incidence of new lung cancer cases in the US was 56 per 100,000 people and the rate of lung cancer death surpassed the rate of any other cancer death with 38.5 per 100,000 people.[2]

Although screening for lung cancer using low dose computed tomography (CT) scanning has been recommended in the US since 2013, the majority of individuals are diagnosed either after seeking clinical care with symptoms or as an incidental finding after imaging.[3] The poor outcomes associated with lung cancer are at least partly the result of the length of time between a patient first experiencing bodily changes and being diagnosed.[4-7] Based on a pooled analysis of 56 studies, the median time from symptom onset to diagnosis ranged from 41 to 143 days.[8] Unfortunately, a significant proportion of individuals with lung cancer are at advanced stages at the time of diagnosis and have an overall survival rate measured in months.[9]

There has been surprisingly little US research on patients' perceptions of the diagnostic pathways for lung cancer. Most research assessing time to diagnosis has been performed in European health care systems and in smokers, making comparisons to the US population or to non-smokers difficult.[10,11] There has been almost no research on the diagnostic experiences of patients with advanced lung cancer who are receiving targeted therapies for oncogenic mutations such as c-ros oncogene 1 (*ROS1*) mutations (1%), anaplastic lymphoma kinase (*ALK*) rearrangements (3%–7%), and epidermal growth factor receptor (*EGFR*) mutations (10%–15%).[12] Targeted therapy has improved outcomes for patients with these mutations, with median overall survival times of 52.1 months for *ROS1*, 81 months for ALK, and 29.7 months for *EGFR*. Thus, understanding the pathway to diagnosis is especially important in this population.[13-16]

The purpose of this study was to explore the experience of the diagnostic process among patients with advanced lung cancer whose tumors tested positive for oncogenic driver mutations in order to identify potential areas to improve the efficiency and experience of the diagnostic pathway.

Methods

Study design: This qualitative study used in-depth individual patient interviews and was approved by the University of Washington Institutional Review Board (Study number STUDY00005438).

Study population: Participants met the following inclusion criteria: (1) histologic or cytologically confirmed diagnosis of metastatic or advanced non-small cell lung cancer (NSCLC) with the presence of one oncogenic alteration (*EGFR, ALK,* or *ROS1*); (2) physically and psychologically well enough to participate; (3) proficient in English; and (4) receiving care in the US. We identified patients using online oncogene-focused lung cancer support groups. Detailed methods are included in a previous publication.[17]

Study procedures: Participants were interviewed by phone, video-conference, or in-person depending on location and preference. One author (MA) conducted the interviews after receiving verbal consent. Interviews were audio-recorded and transcribed verbatim. Participants

For peer review only - http://bmjopen.bmj.com/site/about/guidelines.xhtml

were asked to describe their diagnostic journey from the moment of first noticing symptoms to initial treatment. The interviewer asked follow up questions for clarification. Participants were given a $50 gift card for participating. Interview guide is included in appendix 1.

Analysis: NVIVO 11 was used to organize the data and conduct the analysis. Inductive and deductive thematic analysis was applied. As outlined by Carspecken,[18] the transcripts were read by the lead author (MA) and low-level codes were developed. The codes were then collated by topic. Codes were mapped following the Model of Pathways to Treatment (Figure 1).[8,19,20] Themes and subthemes emerged through an iterative process, and all authors engaged in peer debriefings as groups and dyads reviewing aspects of the work, including coding and analysis, theme development, and description of findings. Themes were organized based on the *Aarhus statement on cancer diagnostic research* stages: patient interval, primary care interval, and secondary care interval.[21,22] Transcripts and themes were reviewed and synthesized to characterize the different types of diagnostic pathways experienced by patients.

MA is a lung cancer patient, family doctor, and qualitative researcher. MLZS is a researcher with experience in qualitative research. MT is a family physician in the US with extensive research experience on disease diagnosis. BHLG is an oncologist and health service researcher. FMW and RDN are primary care lung cancer researchers from the UK. MA did the main analysis and engaged in peer debriefing with co-authors as dyads and groups. Co-authors review aspects of the work, such as analysis and coding, theme development, and writing results.

Patient and Public Involvement: The main author is a stage 4 lung cancer patient and a member of a one lung cancer support groups. The research questions were informed by conversations with lung cancer communities. Patient gatekeepers helped recruiting participants by sharing about the study in their support groups. The study will be shared with cancer communities on social media, and specifically in the support group venues.

Results

A total of 40 patients were interviewed. Their mean age was 48 (range 30–75); 12 were male and 28 were female. Interviews were conducted a median of 19.5 months (range 3–152) after diagnosis (Table 1). All participants had a primary diagnosis of metastatic or advanced NSCLC with one driver oncogenic alteration. We noted seven different diagnostic pathways experienced by patients; rather than simply linear or predictable courses, pathways to diagnosis were more iterative and circular (Figure 2).

A. The Experience of Lung Cancer Diagnosis

Emergent themes within the diagnostic intervals (patient, primary care, and secondary care) are detailed below.

1) Patient interva1 (Table 2)

For peer review only

a) Initial concerns about symptoms despite low perception of risk

Prior to diagnosis, lung cancer did not come to mind for most participants, especially as most were younger and non-smokers. Many believed their healthy lifestyle protected them against such illnesses. In contrast, those who smoked suspected lung cancer from the onset of symptoms. The participants recalled experiencing various new symptoms or a change in persisting symptoms that concerned them. Most reported nonspecific symptoms; some were respiratory in nature, while others related to organs and systems due to metastatic spread (e.g., bone pain) or were constitutional (e.g., fatigue, weight loss). Some recalled the symptoms being present up to few months prior to diagnosis. A minority did not recall any symptoms. Diagnosis occurred after imaging for other reasons, such as an injury or trauma.

b) Attribution of symptoms to other causes, and not always seeking care immediately

Participants initially attributed their symptoms to reasons other than lung cancer. Coughing, for example, was explained by forest fire smoke in the air; back pain was attributed to muscle spasm; fatigue was blamed on depression, and shortness of breath with activities on excessive weight. Even hemoptysis raised concern for tuberculosis as a more likely cause. Many participants did not worry initially because the symptoms were perceived as mild or they felt others had similar symptoms, such as dismissing a cough during flu season. Finally, some people did not have health insurance at the time of early symptoms, and the potential cost of health care services deterred them from seeking help.

c) Changes in severity or nature of symptoms prompting care-seeking actions

Participants expressed experiencing a change in their level of concern prompting them to seek medical attention. Reasons included symptoms getting worse, especially after initially improving; not responding to treatments for other suspected illnesses; symptoms lingering; disruptive pain; symptoms developing in combination; alarming symptoms appearing, such as hemoptysis or significant weight loss; and symptoms affecting quality of life or affecting sleep. Sometimes family members or friends had advised the person to seek care after noticing symptoms.

Most individuals initially visited their primary care providers (PCPs) to get help with their symptoms or to determine the reason for the symptoms that had become concerning. Some first visited urgent care, especially when they encountered delays in accessing a PCP. Some patients who had established relationships with specialists consulted with them first: some complained to their ear, nose and throat doctor about their hemoptysis while others complained to their gastroenterologist about their shortness of breath.

2) Primary care interval (Table 3)

a) Doctors initially treated for illnesses other than lung cancer

Participants described that providers were not alarmed by, or sometimes dismissed, their initial symptoms. For many, the initial course of management was the investigation and treatment of benign etiologies. In some cases, initial investigations supported other diagnoses, such as a respiratory infection from chest X-ray (CXR) or acid reflux confirmed on endoscopy. In other

For peer review only

cases, initial tests were normal. Some patients' symptoms were attributed to and treated as other diseases, for example, a shortness of breath was attributed to underlying asthma and treated with inhalers and steroids. Some patients were referred to specialists, such as physical therapy or orthopedics for musculoskeletal complaints. The wait for specialist appointments sometimes took several weeks. Not infrequently, providers used "safety netting", or contingency plans, such as scheduling return visits, follow-up CXR, and trying other treatment plans.

b) Discovery of imaging abnormality, often on CXR and/or chest CT, leading to diagnosis

A major turning point identified by some participants was getting a CXR, either at their request or prompted by their PCP, intended to identify the cause of symptoms. Imaging studies were also ordered when treatment failed or to assess whether previously-noted radiologic findings had been resolved. Occasionally, imaging tests were used to evaluate incidental conditions such as injuries, while other patients received CXR to follow up on nodules seen on previous imaging. Other imaging tests used to evaluate symptoms elsewhere in the body identified lung cancer as an incidental or unexpected finding, such as magnetic resonance imaging (MRI) for back pain or breast-screening MRIs identifying lung lesions.

For many patients, a diagnosis of lung cancer was supported by a chest CT done after an abnormal CXR or to discover the primary site after a metastasis was found. Scheduling the CT scan was often rushed. Sometimes PCPs pushed for this to happen or, when scheduling was delayed, advised patients to go to the emergency room (ER).

c) Severity of symptoms prompting need for emergency care

Some patients went directly to the ER with distressing symptoms such as severe shortness of breath. Others sought care in the ER for symptoms such as headache and back pain as they had no PCP. At times, the patient's condition deteriorated quickly, requiring admission due to hypoxia or losing consciousness with brain tumors causing seizures. Occasionally, delays in diagnostics or the perception that their PCP could not offer much besides office testing prompted the patient to go to ER. Other patients were advised to go to the ER after findings such as a pulmonary embolism or massive brain metastasis. At the ER, it was not uncommon for the patient to be admitted. Some patients demanded urgent consultations from specialists and to be admitted to complete the cancer workup and start treatment.

3) Specialty care interval (Table 4)

a) The pivotal nature of tissue sample collection

Once imaging raised the alarm for cancer, interventional radiologists, pulmonologists, or thoracic surgeons obtained tissue samples. While some patients saw a specialist fairly quickly, others experienced significant delays. Bronchoscopy, needle biopsies, sampling of pleural effusions, and occasionally surgical biopsies were used to clarify if the lesions seen on imaging were cancer, to identify the type of cancer, and to obtain tumor tissue for genetic testing. Results were delivered within a few days. While a bronchoscopy was often uneventful, it sometimes led to major bleeding, collapsed lungs, or the patient requiring resuscitation. Occasionally, concerns over the procedure led to delays in this diagnostic step. When decisions were made to forego biopsy, patients felt they were provided false reassurance based on less reliable information, such as the appearance on images and their overall assumed low-risk of cancer.

For peer review only

b) Access to oncologists determined staging but perceived delays led to distress

Patients were referred to an oncologist once diagnosed. The referral was made urgently, often by the PCP or pulmonologist based on imaging findings or following pathology results. It was not uncommon for patients to perceive a delay in making appointments, causing frustration. To identify the right specialist and overcome delays, patients often leveraged personal connections or sought help from family and the cancer community. First meetings with oncologists often involved reviewing the results and setting treatment plans. These were usually short, especially if molecular results were not back. Oncologists often completed the diagnostic workup by ordering additional imaging such as positron emission tomography (PET) scans or brain MRIs. Since our participants had advanced diseases, PET scans often showed metastasis outside the lungs.

c) Genetic testing was crucial in directing patients to targeted treatments

For our participants, molecular testing on tissue or blood samples was obviously an instrumental part of their diagnosis. Realization of a positive mutation was met with relief, as patients were fortunate to be a candidate for targeted therapy. However, molecular testing results sometimes took several weeks or were overlooked by providers. Looking back, some patients described frustration at being given chemotherapy instead of waiting for molecular testing results. Some, however, needed emergency chemotherapy, radiation, or surgery to relieve symptoms.

Discussion

As the first on the subject, this study contributes to the literature on pathways to diagnosis and the intervals of diagnosis among patients with advanced lung cancer on targeted therapy. The participants were mostly young, non-smokers, unlike those in previous research in this area. We used a well-established model to map participants' experiences from their initial realization of symptoms, through contact with health care, and diagnostic workup.[19,20]

Previous studies on this 'patient interval' suggested that atypical or vague symptoms caused delays in knowing when to seek care. Previous research (with participants who were predominantly smokers) noted reluctance among patients to visit their health care provider when symptoms emerge,[6] but this pattern was not reported by the majority of our study participants. Because they were younger than the average age at presentation of lung cancer and/or presented with non-specific symptoms,[4] their concerns were typically attributed initially to benign diseases. Recognizing the symptoms and making a diagnosis can be particularly challenging when a patient has comorbid conditions with symptoms similar to those of lung cancer.[4,23]

Many patients perceived inefficiency and delays in the primary care interval. However, these perceptions were made retrospectively, bringing into question whether an actual delay took place. Some patients felt they had to advocate for themselves to obtain initial diagnostic testing and push for more advanced testing when initial tests were inconclusive. This finding is consistent with the role of self-advocacy in improving the quality of care for patients with cancer.[24,25] Previous studies suggested dismissive responses from PCPs may impact patients' decisions to consult care again.[26,27] In contrast, our participants reported persistence and, at times, sought other providers. Some

For peer review only - http://bmjopen.bmj.com/site/about/guidelines.xhtml

providers clearly had contingency and follow-up plans, but other patients felt they were dismissed without clear "safety netting".[28]

Previous US studies of lung cancer patients have suggested delays occur mainly in the primary care interval through misdiagnosis (and from monitoring nodules) rather than in the specialty-care interval.[29] In contrast, difficulty in accessing secondary care is a major cause for delays in the United Kingdom.[6] Our study found that patients' sense of urgency and perception of unnecessary waiting intensified after receiving imaging diagnosing possible cancer. Many complained about delays in accessing pulmonologists, oncologists, or in results from molecular testing. While these waits were were fairly short and probably had little impact on the overall prognosis, they did appear to intensify patient emotion.

This study has many strengths. It is the first to explore the perspectives of a relatively new group of lung cancer survivors: those on targeted therapies that experience significantly superior outcomes. Interviewees may have been better able to reflect on their diagnostic journey in the absence of side effects from chemo or radiation therapy. Our findings were developed within an existing framework used in research on early diagnosis of cancer by many other countries. Our study also has a few limitations. Only a small proportion of our participants experienced barriers in accessing care due to financial concerns, which may have limited our ability to determine the impact of these factors.[29] Our sampling relied on individuals identified from lung cancer survivor groups, which may have reduced the representativeness, particular of individuals from less affluent backgrounds and over-recruited patients who were more engaged with their disease and diagnostic work up. Also, we did not actively seek to define smoking status during the interviews, thus we omitted characterizing the sample by this factor. Finally, recall bias and differential recall bias are major concerns with this type of research.

Our study has important practical implications. First, lung cancer affects everyone, including those thought to be at low risk. The public must be made aware of this so when new symptoms appear, they will seek healthcare promptly. This advice should be tempered with knowledge of the extremely low probability of cancer in most patients and the poor predictive value of most symptoms. Second, PCPs should be vigilant for rare but serious diseases with similar symptom profiles to benign conditions. "Safety netting" should including sharing diagnostic uncertainty and encouraging patients to return for further assessment when symptoms fail to respond. More precise diagnostic tools would be valuable to PCPs in this difficult task, but ready access to CXR and CT is clearly important. Third, while access to secondary care for serious conditions like cancer may not be a challenge for all patients in the US, the need for coordinating care, communication with patients, and provision of up-to-date standards of practice continue to be an issue.

For peer review only

Acknowledgements: The authors would thank LUNGevity, especially Upal Basu Roy, for helping connect with patient advocacy and support groups. They also thank patients and patient advocates Janet Freeman-Daily, Jill Feldman, Ivy Elkins and Tom Carroll, for helping connect us to research participants. They also acknowledge the ROSOneder support group, the ALK-Positive Facebook Support Group and the EGFR Resisters for supporting and promoting this work.

Funding: This research is linked to the CanTest Collaborative, which is funded by Cancer Research UK [C8640/A23385], of which author Fiona Walter is director and authors Richard Neal and Matthew Thompson are associate directors and Co-investigator. Information, conclusions and opinions expressed in this presentation are of the authors and no endorsement is intended or should be inferred.

Competing Interests: The Authors have no conflict of interest to report.

Contribution Statement: MA, MLZS, FMW, RDN, BHLG, MT contributed to the literature review and the conceptualization of the work. MA conducted the interviews. MA conducted the primary analysis of the data. MLZS and MT did peer debriefing and review of analysis with MA individually and in groups. MA, MLZS, FMW, RDN, BHLG, MT all contributed to the writing of the discussion. All the authors reviewed and approved the final version of the study.

Data sharing: Deidentified data will be shared upon request.

For peer review only

References

1. Siegel RL, Miller KD, Jemal A. Cancer statistics, 2017. CA Cancer J Clin. 2017;67(1):7-30.
2. Center for Disease Control and Prevention. Leading Cancer Cases and Deaths, All Races/Ethnicities, Male and Female, 2017. Available at https://gis.cdc.gov/Cancer/USCS/DataViz.html accessed 6/20/2020.
3. Latimer KM, Mott TF. Lung cancer: diagnosis, treatment principles, and screening. *Am Fam Physician*. 2015;91(4):250–256.
4. Dobson C, Russell A, Brown S, Rubin G. The role of social context in symptom appraisal and help-seeking among people with lung or colorectal symptoms: A qualitative interview study. Eur J Cancer Care (Engl). 2018 Feb 8. doi: 10.1111/ecc.12815. [Epub ahead of print] PubMed PMID: 29419943.
5. McLachlan S, Mansell G, Sanders T, Yardley S, van der Windt D, Brindle L, Chew-Graham C, Little P. Symptom perceptions and help-seeking behaviour prior to lung and colorectal cancer diagnoses: a qualitative study. Fam Pract. 2015 Oct;32(5):568-77. doi: 10.1093/fampra/cmv048. Epub 2015 Jun 22. PubMed PMID: 26099812; PubMed Central PMCID: PMC4576759.
6. Neal RD, Robbé IJ, Lewis M, Williamson I, Hanson J. The complexity and difficulty of diagnosing lung cancer: findings from a national primary-care study in Wales. Prim Health Care Res Dev. 2015 Sep;16(5):436-49. doi: 10.1017/S1463423614000516. Epub 2014 Dec 8. PubMed PMID: 25482333.
7. Walter FM, Rubin G, Bankhead C, Morris HC, Hall N, Mills K, Dobson C, Rintoul RC, Hamilton R. Emeryet J. Symptoms and other factors associated with time to diagnosis and stage of lung cancer: a prospective cohort study. *Brit J Cancer*, 2015 Mar 31;112:S6-S13. doi: 10.1038/bjc.2015.30.
8. Jacobsen MM, Silverstein SC, Quinn M, et al. Timeliness of access to lung cancer diagnosis and treatment: A scoping literature review. Lung Cancer. 2017;112:156-164.
9. Goldstraw P, Chansky K, Crowley J, Rami-Porta R, Asamura H, Eberhardt WE, Nicholson AG, Groome P, Mitchell A, Bolejack V. The IASLC Lung Cancer Staging Project: Proposals for revision of the TNM stage groupings in the forthcoming (eighth) edition of the TNM classification for lung cancer. J Thorac Oncol. 2016;11(1):39-51
10. Vinas F, Ben Hassen I, Jabot L, Monnet I, Chouaid C. Delays for diagnosis and treatment of lung cancers: a systematic review. Clin Respir J. 2016;10(3):267-271.
11. Olsson JK, Schultz EM, Gould MK. Timeliness of care in patients with lung cancer: a systematic review. Thorax. 2009;64(9):749-756.
12. Lovly C, Horn L, Pao W. Molecular profiling of lung cancer, My Cancer Genomelast updated March 16, 2018. Available: https://www.mycancergenome.org/content/disease/lung-cancer/
13. Korpanty GJ, Graham DM, Vincent MD, et al. Biomarkers that currently affect clinical practice in lung cancer: EGFR, ALK, Met, ROS-1, and KRAS. Front Oncol 2014;4:204.doi:10.3389/fonc.2014.00204
14. Okamoto I, Morita S, Tashiro N, et al. Real world treatment and outcomes in EGFR mutation-positive non-small cell lung cancer: long-term follow-up of a large patient cohort. Lung Cancer 2018;117:14–19.doi:10.1016/j.lungcan.2018.01.005

For peer review only

15. Park S, Ahn B-C, Lim SW, et al. Characteristics and outcome of ROS1-positive non-small cell lung cancer patients in routine clinical practice. J Thorac Oncol 2018;13:1373–82.doi:10.1016/j.jtho.2018.05.026.
16. Pacheco JM, Gao D, Smith D, et al. Natural history and factors associated with overall survival in stage IV ALK-rearranged non-small cell lung cancer. J Thorac Oncol 2019;14:691–700.doi:10.1016/j.jtho.2018.12.014.
17. Al Achkar M, Marchand L, Thompson M, Chow LQ, Revere D, Baldwin LM. Unmet needs and opportunities for improving care for patients with advanced lung cancer on targeted therapies: a qualitative study. BMJ open. 2020 Mar 1;10(3):e032639.
18. Carspecken PF. Critical ethnographies from Houston: Distinctive features and directions. Critical ethnography and education. 2001;5:1-26.
19. Walter FM, Scott SE, Webster A, Emery JD. The Andersen Model of Total Patient Delay: a systematic review of its application in cancer diagnosis. J Health Services Research & Policy 2012;17(2):110-118
20. Scott SE, Walter FM, Webster A, Emery JD. The model of pathways to treatment: conceptualization and integration with existing theory. Br J Health Psychol, 2013; 18(1):45-65.
21. Weller D, Vedsted P, Rubin G, Walter F, Emery J, Scott S, Campbell C, Andersen RS, Hamilton W, Olesen F, Rose P, Nafees S, van Rijswijk E, Muth C, Beyer M, Neal RD. The Aarhus Statement: Improving design and reporting of studies on early cancer diagnosis. *BJC* 2012,**106**:1262-1267. DOI 10.1038/bjc.2012.68
22. Coxon, D., Campbell, C., Walter, F.M. *et al.* The Aarhus statement on cancer diagnostic research: turning recommendations into new survey instruments. *BMC Health Serv Res* **18,** 677 (2018). https://doi.org/10.1186/s12913-018-3476-0
23. Carter-Harris L, Hermann CP, Draucker CB. Pathways to a lung cancer diagnosis. J Am Assoc Nurse Pract. 2015 Oct;27(10):576-83. doi: 10.1002/2327-6924.12242. Epub 2015 Mar 12. PubMed PMID: 25772906; PubMed Central PMCID: PMC4567975.
24. Mathews M, Bulman D, Ryan D. The role of patient and physician advocacy in reducing wait times for cancer care: a qualitative analysis. CMAJ open. 2017 Oct;5(4):E773.
25. Jørgensen CR, Thomsen TG, Ross L, et al. What Facilitates "Patient Empowerment" in Cancer Patients During Follow-Up: A Qualitative Systematic Review of the Literature. Qual Health Res 2018;28:292–304.
26. Birt L, Hall N, Emery JD, Banks J, Mills K, Johnson M, Hamilton W, Walter FM. Responding to symptoms suggestive of lung cancer: a qualitative interview study. BMJ Open Respir Res, 2014;1:e000067. doi:10.1136/bmjresp-2014-000067.
27. Walter FM, Birt L, Cavers D, Scott S, Emery J, Burrows N, Cavanagh G, MacKie R, Weller D, Campbell C. 'This isn't what mine looked like': a qualitative study of symptom appraisal and help seeking in people recently diagnosed with melanoma. BMJ Open, 2014;4(7):e005566. doi: 10.1136/bmjopen-2014-005566.
28. Nicholson BD, Mant D, Bankhead C. Can safety-netting improve cancer detection in patients with vague symptoms?. Bmj. 2016 Nov 9;355:i5515.
29. Vidaver RM, Shershneva MB, Hetzel SJ, Holden TR, Campbell TC. Typical time to treatment of patients with lung cancer in a multisite, US-based study. Journal of oncology practice. 2016 Jun;12(6):e643-53.

For peer review only

Table 1. Participant characteristics.

Table 2. Supportive quotes for the.

Table 3. Supportive quotes for the.

Table 4. Supportive quotes for the.

Figure 1. The conceptual model of pathway to treatment.

Figure 2. Identified pathways to diagnosis.

Appendix 1. Interview guide.

For peer review only

Table 1. Participant characteristics.

Participant Characterestics	Median (Range) / Count
Age	49 (30-75) years
Gender	
Male	12
Female	28
Race	
White	34
Others (Asian, Hispanic, biracial (Asian and Hispanic))	6
Region in the US	
West	18
Northeast	8
Midwest	7
South	6
Insurance	
Private	34
Medicare	4
Medicaid	2
Time since diagnosis	19.5 (3-152) months
Cancer Stage at Time of Interview	
IV	38
IIIb	2
Mutation	
ALK	20
EGFR	14
Ros1	6

For peer review only

Table 2. Supportive quotes for the patient interval.

**Initial concerns about symptoms despite low perception of risk**
I have not been into a doctor for a medical check-up at all in all that time. I never had any days off taken my entire work experience. (1001)<br>*I looked really healthy and I'm not a smoker. (3005)*<br>*I started seeing symptoms three-four months before diagnosis. I noticed some tightness in my chest. (1003)*<br>*I just had a dry cough that would not go away. (2007)*
**Attribution of symptoms to other causes, and not always seeking care immediately**
There were a lot of forest fires. The air was always really smoky and I thought maybe part of the headaches or not feeling quite right was caused by the smoke. (2013)<br>*I was having some lower back pain in the kidney area and had some other symptoms that made me think maybe I have got kidney stones. (2006)*<br>*Everybody else in the family also seemed to have flu- like illness going on with a cough; cold-cough kind of thing.(1005)*<br>*I was very weak, very lethargic; the worst I ever felt in my life. I tried to self-medicate. I was not insured. (1003)*
**Changes in severity or nature of symptoms prompting care-seeking actions**
Three more weeks went by and the cough continued to get worse to the point where my chest started hurting and I had a little bit of a backache. (1005)<br>*My wife came back from China, she was away for about a month. She said, "Your coughing is different." At the time, I didn't notice anything yet. (2012)*<br>*I coughed a little blood. I am not stupid I knew I had big trouble. There was no question; I called the doctor. (1012)*<br>*I decided, I'm going to go ahead and see my primary care physician to see if maybe she had some more suggestions of what I can do to help this throat situation. (1017)*

For peer review only

Table 3. Supportive quotes for the primary care provider interval.

**Doctors initially treated for illnesses other than lung cancer**
I recall going to see the Primary Care Physician and mentioned, "I'm constantly clearing my throat." They casually dismissed me; the symptom continued. (3002)<br>*I went to the doctor and she did full blood work and said everything looks great. She said the cough was probably just a little bit of a remnant from the cold and typically it can take 3, 4, 5 or even 6 months to go away and not to worry about it too much. (1001)*<br>*I went back to my doctor again and said, "okay, we've tried asthma, we've tried the allergy, here is some reflux medications," which kind of helped. She sent me to my doctor that specializes in reflux. We did an endoscopy. They came back with, "you do have reflux." (3004)*<br>*I kept seeing various doctors and they would always send me home. Like, "Oh, it's a seasonal cold. Oh, it's allergies. Oh, you pulled the muscles from coughing too much, here are some steroids." (1008)*<br>*I went to a walk in clinic two different times and was diagnosed with walking pneumonia. Both of those times, I did have an x-ray of the chest, and it just showed some cloudiness, it didn't show any kind mass. (2007)*<br>*She put me on a different prescription but she said, "If you're not better in a couple of weeks, come back and we'll do a full pulmonary workup and we'll do more diagnostic testing 'cause this was concerning." (3001)*
**Discovery of imaging abnormality, often on CXR or a chest CT led to diagnosis**
The doctor gave me steroids was leaving the room, I said something to the effect of, "I thought I would have to get an X-ray." I'm the one who mentioned the word, "X-ray". (1017)<br>*I went to get an x-ray of my left rib cage. It felt like something was there. I told my doctor that I think I have cancer and I want her to check for cancer. So she obliged. (1009)*<br>*I made an appointment and set me for a chest x-ray. And this is was to me really an important point. There was a radiologist sitting in the booth. He looked at me and from the look on his face I just knew. (1011)*<br>*After the car accident I was taken to a trauma center and they scanned me and said, "You have a broken back and lung cancer." (2009)*<br>*I went back to the doctor the next day and she took a look and she said, "Hmm, I don't like that (swelling in supra-clavicular area)." And she sent me for an ultrasound. (3001)*<br>*I went for a physical to my primary care doctor. He noticed that I had motor deficits in my hands. He suggested that I get an MRI. I actually had to go and see a neurologist in order to get the prescription for an MRI and paid for. (2013)*<br>*As soon as the order went in for the chest x-ray, I went in to have it done. That night my doctor called back and said, "we saw some things on the chest x-ray, we want to get you in for CT scan." So the chest x-ray was a Monday, the CT scan was a Thursday. On the night of the CT scan, she called back and said, "It looks like cancer." (3004)*<br>*She noticed that my breath sounds weren't right. So she ordered a CT and called me the next day and told me that she was going to send me for a PET. She was pretty concerned that it was lung cancer. (1004)*
**Severity of symptoms prompting need for emergency care**
I was scheduled for a CT scan but the next opening wasn't for like 2 or 3 weeks. I was having so much coughing that I couldn't speak or breathe properly. So I called my APNP's office. She advised that I should go to the ER and get a CT scan. (2007)<br>*We scheduled the biopsy for Thursday. Tuesday morning before I could go for the biopsy, I woke up coughing up blood, a considerable amount of blood which was new that it never happened. So I drove myself to the ER*<br>*The second I went in the pulmonologist office, he checked my oxygen and it was 85%. I took his advise and went to the hospital. (1014)*

For peer review only

Table 4. Supportive quotes for the secondary care interval.

**The pivotal nature of tissue sample collection**
The PCP said, "I think you have a problem. You got to go and see a Pulmonologist immediately." Finding a Pulmonologist with an opening is impossible. (3002)<br>*She said it looks like a metastatic disease. She set me up with a biopsy of the lung and a biopsy of the liver. (3003)*<br>*I tried to have a lung biopsy done and I was sitting on the table and the radiologist came in and he said, "I can't biopsy that nodule, no way." The team were all arguing about it over me and finally the radiologist said it is not biopsiable and so I left. They said, well, that probably is not cancer. (1011)*<br>*I had a biopsy of the lungs and ended up with a completely collapsed lung and a chest tube. (1006)*<br>*A senior pulmonologist said, "We suggest drain her lung, drain her effusion and let her out." But the hospitalist was like, "I don't want to let her out until we get a biopsy because she's going to be in the community and it's trying to schedule all of these and she's going to be given and run around and this is an emergent case so I'm leaving her until she can get the biopsy." (1019)*<br>*I had a needle biopsy and he called me a couple of days later, "It's lung cancer and it's adenocarcinoma, and I'm going to send you to an oncologist. (3001)*
**Access to oncologists determined staging but perceived delays led to distress**
I was discharged from the hospital, came home, had a follow-up appointment a few days later with an oncologist who was just part of the healthcare system. They just assigned to me to somebody. (2008)<br>*I was leaving ever more frantic messages and calling again and again and pushing the reception desk. It was about quarter to 12 before I finally convinced her I needed to talk with the doctor today rather than wait, find out how long I might live. (2014)*<br>*I was able to find a lung cancer foundation. And one of the folks there told me about a lung cancer oncologist in an area close to me and said, "You should reach out to them. And tell them I told you to give them a call." And so I did just that. And the doctor called me back. (3002)*<br>*They noted that there were tumors spotted on in my neck region and at that point in time, they wanted to do a full PET scan to figure out what the extent it was. They turned around really quickly. I must say after the original scan, the quickness of my treatment and exploratory work was very fast. (1013)*<br>*I had developed what I had thought was sciatica, but when they did the scan they found out that it was metastasis in my bone that was hitting my sacrum that was kind of causing the sciatic nerve to be inflamed.(2001)*
**Genetic testing was crucial in directing patients to targeted treatments**
I'm grateful my oncologist ordered molecular testing. I know that's becoming standard as care now, it was not quite so much standard as care at that stage. (1001)<br>*So the following week we are supposed to have an appointment but the insurance took a while to approve everything. We postponed that appointment till we got result and the result were ALK positive. (1019)*<br>*When week number 4 went around, (the local oncologist) has not been following up with me. I've been calling you, we still don't have results. I was uncomfortable right around week 6, so I flew and sat down with an oncologist (at a major cancer center) and he basically said well we don't need to wait. Let's do blood test (liquid biopsy). I'll have the results for you in 7 days. (1020)*<br>*I think that somebody dropped the ball at the hospital because the request for the testing wasn't sent until three weeks after they did the surgery they hadn't even requested to do the molecular testing. So when they finally did it still took another few weeks. (1018)*<br>*I had a week of radiation and they were still waiting for the mutation to come back. (3006)*<br>*He wanted me to start chemo treatment immediately because it seemed to be very aggressive whatever this was. Without waiting for the results of any genomic testing and this is still a point of concern for me because, looking back, I feel things work done improperly. We did not wait for the results of the genomic testing. I was started on chemotherapy. (2008)*<br>*The surgery basically gave me a hug and said, there's some really good news, the tumors tested positive for ROS 1, and I had no idea what this means. (3002)*<br>*I'll never forget when my doctor came in and he said, "Hey, you have the ALK mutation." And he said, "You're really lucky." And I'm like, "What do you mean? How am I lucky?" And he was like, "We have this great medicine that was just approved by the FDA. (1008)*

For peer review only

For peer review only

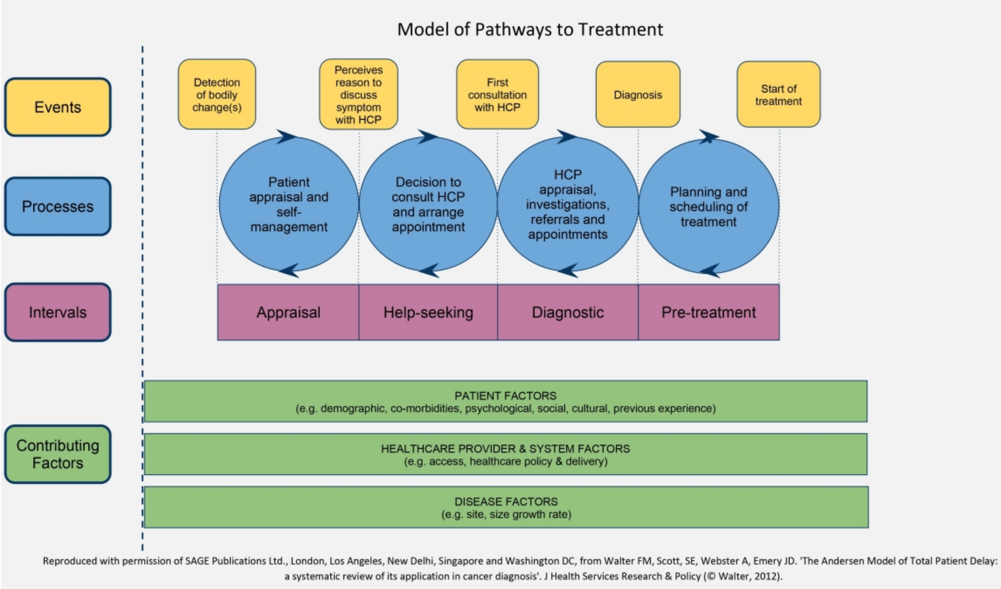

Figure 1. The conceptual model of pathway to treatment.

320x188mm (300 x 300 DPI)

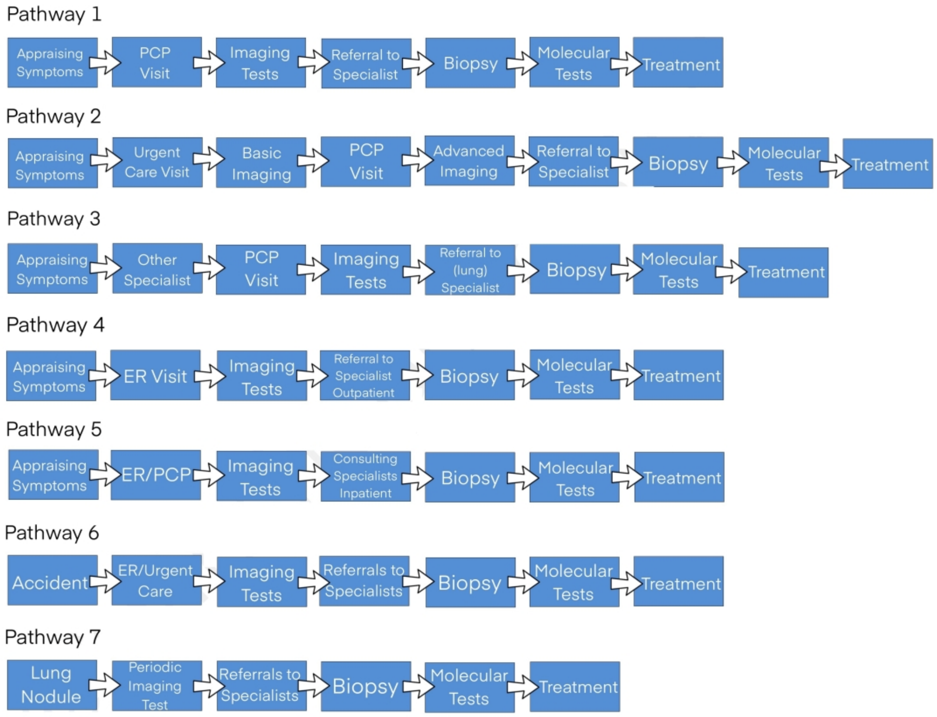

Figure 2. Identified pathways to diagnosis.

670x548mm (132 x 132 DPI)

Interview Protocol

First Topic Domain: Life Before Cancer Diagnosis

- *Lead-off question*

"*I want to know about your life before cancer diagnosis. Think about the time back then and tell me about typical days of your life. Pretend that you are telling your life story to a friend. I want to know everything, family, school, work, friends, hobbies.*"

- Covert categories: [day to day life; meaning-making; identity; self-image; what did the person do before; who the person was before; aspects of life relevant to the person; norms and values; education; how the person looks at oneself in the past; how much reconstruction is taking place; the tone of feeling when reflecting about the past; the relation to the old self; others.]
- *Possible follow-up questions*

1. *Tell me about you and your family before*
2. *Tell me about significant other(s)*
3. *Tell me what you did for work today. Describe your job before.*
4. *Tell me about what you did in your leisure time (friends, hobbies, etc.)*

Second Topic Domain: Diagnosis of Cancer

- *Lead-off question*

"*Now I want to learn about your cancer itself. Tell me the story of your cancer diagnosis and treatment.*"

- Covert categories: [the experience of early symptoms; the internal dialogues and making decision to seek help; the experience of making the diagnosis; the role of family and friends; the experience with healthcare; perceptions about doctors, nurses, and staff; opinions of the health system at large; the decisions around treatment; the treatment; side effects; others]
- *Possible follow-up questions*

1. *How did the disease present itself?*
2. *How was your experience with the doctors, hospitals, clinic staff?*
3. *How was your experience with treatment?*
4. *How did you and your doctor make decisions about treatment?*

Third Topic Domain: Life after Cancer

- *Lead-off question*

For peer review only

"*Now I want to learn about your life after cancer diagnosis. Tell me about your life now, the day to day life. Walk me through a typical day of your week.*"

- Covert categories: [how is the patient with cancer living life. What is different from before; what is the same; work; school; family; relationships; emotions/feelings; desires; struggles; things that are going well; things that are not going well; resilience; others]
- *Possible follow-up questions*

1. *How do you spend your time if not working?*
2. *What are non-cancer related things you do on day to day?*
3. *What changed from before?*

Fourth Topic Domain: Coping with Cancer

- *Lead-off question*

"*I want to focus now on what you are doing to cope with cancer. What are you doing on day-to-day basis to deal with cancer? Tell me everything in the area of health and wellbeing you are doing related to dealing with cancer. [if there are special treatment days] tell me about the treatment days.*"

- Covert categories: [health related actions; exercise; diet; taking medications; other categories the patient considers relevant; why are they doing every one; what are implicit theories behind the workings of these actions; support persons]
- *Possible follow-up questions*

1. *What are some things that you are doing to live better/be healthier?*
2. *What are some things you are doing to get better at dealing with cancer?*
3. *What have you found helpful?*
4. *How do you get strength?*
5. *How do you find meaning?*

Fifth Topic Domain: Unmet Needs

- *Lead-off question*

"*What is it that you need today to make things better in your day to day. I am speaking about the emotional need, physical need, and spiritual needs and any others.*"

- Covert categories: [unmet needs; desires; wants; struggles; conflicts; limitations; perceptions of what can be helpful; perceptions of what is contributing to the person's struggle; how can others help the person; how can the person help herself; others]
- *Possible follow-up questions*

1. *What could improve your quality of life today?*

For peer review only

2. *What do you need for your emotional wellbeing?*

3. *What is it that can be done for you so you feel better health-wise?*

4. *What is it that can be done to improve your experience with your healthcare team?*

For peer review only

Standards for Reporting Qualitative Research (SRQR)*

http://www.equator-network.org/reporting-guidelines/srqr/

Title and abstract

	Page/line no(s).
**Title** - Concise description of the nature and topic of the study Identifying the study as qualitative or indicating the approach (e.g., ethnography, grounded theory) or data collection methods (e.g., interview, focus group) is recommended	1
**Abstract** - Summary of key elements of the study using the abstract format of the intended publication; typically includes background, purpose, methods, results, and conclusions	3

Introduction

**Problem formulation** - Description and significance of the problem/phenomenon studied; review of relevant theory and empirical work; problem statement	5
**Purpose or research question** - Purpose of the study and specific objectives or questions	5

Methods

**Qualitative approach and research paradigm** - Qualitative approach (e.g., ethnography, grounded theory, case study, phenomenology, narrative research) and guiding theory if appropriate; identifying the research paradigm (e.g., postpositivist, constructivist/ interpretivist) is also recommended; rationale**	6
**Researcher characteristics and reflexivity** - Researchers' characteristics that may influence the research, including personal attributes, qualifications/experience, relationship with participants, assumptions, and/or presuppositions; potential or actual interaction between researchers' characteristics and the research questions, approach, methods, results, and/or transferability	6
**Context** - Setting/site and salient contextual factors; rationale**	5
**Sampling strategy** - How and why research participants, documents, or events were selected; criteria for deciding when no further sampling was necessary (e.g., sampling saturation); rationale**	5-6
**Ethical issues pertaining to human subjects** - Documentation of approval by an appropriate ethics review board and participant consent, or explanation for lack thereof; other confidentiality and data security issues	5
**Data collection methods** - Types of data collected; details of data collection procedures including (as appropriate) start and stop dates of data collection and analysis, iterative process, triangulation of sources/methods, and modification of procedures in response to evolving study findings; rationale**	5-6

For peer review only - http://bmjopen.bmj.com/site/about/guidelines.xhtml

For peer review only

**Data collection instruments and technologies** - Description of instruments (e.g., interview guides, questionnaires) and devices (e.g., audio recorders) used for data collection; if/how the instrument(s) changed over the course of the study	5-6, appendix
**Units of study** - Number and relevant characteristics of participants, documents, or events included in the study; level of participation (could be reported in results)	6, table 1
**Data processing** - Methods for processing data prior to and during analysis, including transcription, data entry, data management and security, verification of data integrity, data coding, and anonymization/de-identification of excerpts	6
**Data analysis** - Process by which inferences, themes, etc., were identified and developed, including the researchers involved in data analysis; usually references a specific paradigm or approach; rationale**	6
**Techniques to enhance trustworthiness** - Techniques to enhance trustworthiness and credibility of data analysis (e.g., member checking, audit trail, triangulation); rationale**	6

Results/findings

**Synthesis and interpretation** - Main findings (e.g., interpretations, inferences, and themes); might include development of a theory or model, or integration with prior research or theory	6-9
**Links to empirical data** - Evidence (e.g., quotes, field notes, text excerpts, photographs) to substantiate analytic findings	tables 2-4

Discussion

**Integration with prior work, implications, transferability, and contribution(s) to the field -** Short summary of main findings; explanation of how findings and conclusions connect to, support, elaborate on, or challenge conclusions of earlier scholarship; discussion of scope of application/generalizability; identification of unique contribution(s) to scholarship in a discipline or field	9-10
**Limitations** - Trustworthiness and limitations of findings	10

Other

**Conflicts of interest** - Potential sources of influence or perceived influence on study conduct and conclusions; how these were managed	11
**Funding** - Sources of funding and other support; role of funders in data collection, interpretation, and reporting	11

*The authors created the SRQR by searching the literature to identify guidelines, reporting standards, and critical appraisal criteria for qualitative research; reviewing the reference lists of retrieved sources; and contacting experts to gain feedback. The SRQR aims to improve the transparency of all aspects of qualitative research by providing clear standards for reporting qualitative research.

For peer review only - http://bmjopen.bmj.com/site/about/guidelines.xhtml

**The rationale should briefly discuss the justification for choosing that theory, approach, method, or technique rather than other options available, the assumptions and limitations implicit in those choices, and how those choices influence study conclusions and transferability. As appropriate, the rationale for several items might be discussed together.

Reference:

O'Brien BC, Harris IB, Beckman TJ, Reed DA, Cook DA. **Standards for reporting qualitative research: a synthesis of recommendations.** *Academic Medicine*, Vol. 89, No. 9 / Sept 2014 DOI: 10.1097/ACM.0000000000000388

For peer review only

For peer review only - http://bmjopen.bmj.com/site/about/guidelines.xhtml

BMJ Open

Experiences along the diagnostic pathway for patients with advanced lung cancer in the United States: A Qualitative Study

Journal:	*BMJ Open*
Manuscript ID	bmjopen-2020-045056.R1
Article Type:	Original research
Date Submitted by the Author:	15-Feb-2021
Complete List of Authors:	Al Achkar, Morhaf; University of Washington, Family Medicine ;<br>Zigman Suchsland, Monica; University of Washington,<br>Walter, Fiona; University of Cambridge, Dept of Public Health and Primary Care<br>Neal, Richard; University of Leeds,<br>Goulart, BH; University of Washington,<br>Thompson, Matthew; University of Washington, Department of Family Medicine
**Primary Subject Heading**:	Oncology
Secondary Subject Heading:	General practice / Family practice
Keywords:	Respiratory tract tumours < ONCOLOGY, QUALITATIVE RESEARCH, Cancer genetics < GENETICS

SCHOLARONE™
Manuscripts

I, the Submitting Author has the right to grant and does grant on behalf of all authors of the Work (as defined in the below author licence), an exclusive licence and/or a non-exclusive licence for contributions from authors who are: i) UK Crown employees; ii) where BMJ has agreed a CC-BY licence shall apply, and/or iii) in accordance with the terms applicable for US Federal Government officers or employees acting as part of their official duties; on a worldwide, perpetual, irrevocable, royalty-free basis to BMJ Publishing Group Ltd ("BMJ") its licensees and where the relevant Journal is co-owned by BMJ to the co-owners of the Journal, to publish the Work in this journal and any other BMJ products and to exploit all rights, as set out in our licence.

The Submitting Author accepts and understands that any supply made under these terms is made by BMJ to the Submitting Author unless you are acting as an employee on behalf of your employer or a postgraduate student of an affiliated institution which is paying any applicable article publishing charge ("APC") for Open Access articles. Where the Submitting Author wishes to make the Work available on an Open Access basis (and intends to pay the relevant APC), the terms of reuse of such Open Access shall be governed by a Creative Commons licence – details of these licences and which Creative Commons licence will apply to this Work are set out in our licence referred to above.

Other than as permitted in any relevant BMJ Author's Self Archiving Policies, I confirm this Work has not been accepted for publication elsewhere, is not being considered for publication elsewhere and does not duplicate material already published. I confirm all authors consent to publication of this Work and authorise the granting of this licence.

For peer review only

Experiences along the diagnostic pathway for patients with advanced lung cancer in the United States: A Qualitative Study

Morhaf Al Achkar, MD, PhD

(Corresponding Author)

University of Washington

alachkar@uw.edu

Phone: (206) 520-2405

FAX: (206) 520-2450

331 NE Thornton Place

Seattle, WA, 98125, USA

Monica L Zigman Suchsland, MPH

University of Washington

Fiona M Walter, MA, MD, FRCGP

The Primary Care Unit, Department of Public Health & Primary Care, University of Cambridge, UK

Richard D Neal, PhD FRCGP

University of Leeds

Bernardo H.L. Goulart, MD, MS

University of Washington

Fred Hutchinson Cancer Research Center

Matthew Thompson, MBChB, MPH, DPhil

University of Washington

For peer review only

Word Count: 2988

Number of:

Tables: 4

Figures: 2

Appendix: 1

Keywords: lung cancer, cancer diagnosis, oncognetic alterations.

For peer review only

List of Abbreviations:

CT	Computed Tomography
ROS1	c-Ros oncogene 1
ALK	Anaplastic lymphoma kinase
EGFR	Epidermal growth factor receptor
NSCLC	Non-small cell lung cancer
PCP	Primary care provider
CXR	Chest X-ray
ER	Emergency room
PET	positron emission tomography

Abstract (248 words)

Background: Most lung cancer patients are diagnosed at advanced stages. But the advent of oral targeted therapies improved prognosis of many lung cancer patients.

Purpose: We aimed to understand the diagnostic experiences of patients with advanced lung cancer with oncogenic mutations.

Methods: Qualitative interviews with patients with advanced or metastatic non-small cell lung cancer with oncogenic alterations. Patients were recruited from online support groups within the US. Interviews were conducted remotely or in person. Analysis used an iterative inductive and deductive process. Themes were mapped to the Model of Pathways to Treatment..

Results: 40 patients (12 male and 28 female) with a median age of 48.We identified 9 distinct themes. During the "patient interval," individuals became concerned about symptoms, but often attributed them to other causes. Prolonged or more severe symptoms prompted care seeking. During the "primary care interval," doctors initially treated for illnesses other than cancer. Discovery of an imaging abnormality was a turning point in diagnostic pathwaies. Occasionally, severity of symptoms prompted patients to seek emergency care. During the "secondary care interval," obtaining tissue samples was pivotal in confirming diagnosis. Delays in accessing oncology care sometimes led to patient distress. Obtaining genetic testing was crucial in directing patients to receive targeted treatments.

Conclusions: Patients experienced multiple different routes to their diagnosis. Some patients perceived delays, inefficiencies, and lack of coordination which could be distressing. Shifting the stage of diagnosis of lung cancer to optimize the impact of targeted therapies will require concerted efforts in early detection.

For peer review only

Strengths and limitations of this study

The study's strengths include exploring the perspectives on the diagnosis journey of a large number of participants representing a relatively new group of lung cancer survivors: those on targeted therapies that experience significantly superior outcomes.

Our findings were developed within an existing theoretical framework used in research on early cancer diagnosis by many other countries.

The study's limitations include relying on individuals identified from lung cancer survivor groups, which may have reduced the representativeness, particularly of individuals from less affluent backgrounds.

Only a small proportion of our participants experienced barriers in accessing care due to financial concerns, which may have limited our ability to determine these factors' impact.

Recall bias and differential recall bias are major concerns with this type of research.

For peer review only

Background

Lung cancer is the leading cause of cancer death and the second most common cancer type in the United States (US).[1] In 2016, incidence of new lung cancer cases in the US was 56 per 100,000 people and the rate of lung cancer death surpassed the rate of any other cancer death with 38.5 per 100,000 people.[2]

Although screening for lung cancer using low dose computed tomography (CT) scanning has been recommended in the US since 2013, the majority of individuals are diagnosed either after seeking clinical care with symptoms or as an incidental finding after imaging.[3] The poor outcomes associated with lung cancer are at least partly the result of the length of time between a patient first experiencing bodily changes and being diagnosed.[4-7] Based on a pooled analysis of 56 studies, the median time from symptom onset to diagnosis ranged from 41 to 143 days.[8] Unfortunately, a significant proportion of individuals with lung cancer are at advanced stages at the time of diagnosis and have an overall survival rate measured in months.[9]

There has been surprisingly little US research on patients' perceptions of the diagnostic pathways for lung cancer. Most research assessing time to diagnosis has been performed in European health care systems and in smokers, making comparisons to the US population or to non-smokers difficult.[10,11] There has been almost no research on the diagnostic experiences of patients with advanced lung cancer who are receiving targeted therapies for oncogenic mutations such as c-ros oncogene 1 (*ROS1*) mutations (1%), anaplastic lymphoma kinase (*ALK*) rearrangements (3%–7%), and epidermal growth factor receptor (*EGFR*) mutations (10%–15%).[12] Targeted therapy has improved outcomes for patients with these mutations, with median overall survival times of 52.1 months for *ROS1*, 81 months for ALK, and 29.7 months for *EGFR*. Thus, understanding the pathway to diagnosis is especially important in this population.[13-16]

The purpose of this study was to explore the experience of the diagnostic process among patients with advanced lung cancer whose tumors tested positive for oncogenic driver mutations in order to identify potential areas to improve the efficiency and experience of the diagnostic pathway.

Methods

Study design: This qualitative study used in-depth individual patient interviews and was approved by the University of Washington Institutional Review Board (Study number STUDY00005438).

Study population: Participants met the following inclusion criteria: (1) histologic or cytologically confirmed diagnosis of metastatic or advanced non-small cell lung cancer (NSCLC) with the presence of one oncogenic alteration (*EGFR, ALK,* or *ROS1*); (2) physically and psychologically well enough to participate; (3) proficient in English; and (4) receiving care in the US. We identified patients using online oncogene-focused lung cancer support groups. Detailed methods are included in a previous publication.[17]

Study procedures: Participants were interviewed by phone, video-conference, or in-person depending on location and preference. One author (MA) conducted the interviews after receiving verbal consent. Interviews were audio-recorded and transcribed verbatim. Participants

For peer review only - http://bmjopen.bmj.com/site/about/guidelines.xhtml

were asked to describe their diagnostic journey from the moment of first noticing symptoms to initial treatment. The interviewer asked follow up questions for clarification. Participants were given a $50 gift card for participating. Interview questions and follow up prompts are included in appendix 1.

Analysis: NVIVO 11 was used to organize the data and conduct the analysis. Inductive and deductive thematic analysis was applied. As outlined by Carspecken,[18] the transcripts were read by the lead author (MA) and low-level codes were developed. The codes were then collated by topic. Codes were mapped following the Model of Pathways to Treatment (Figure 1).[8,19,20] Themes and subthemes emerged through an iterative process, and all authors engaged in peer debriefings as groups and dyads reviewing aspects of the work, including coding and analysis, theme development, and description of findings. Themes were organized based on the *Aarhus statement on cancer diagnostic research* stages: patient interval, primary care interval, and secondary care interval.[21,22] Transcripts and themes were reviewed and synthesized to characterize the different types of diagnostic pathways experienced by patients.

MA is a stage 4, ALK positive lung cancer patient, family doctor, and qualitative researcher. MLZS is a researcher with experience in qualitative research. MT is a family physician in the US with extensive research experience on disease diagnosis. BHLG is an oncologist and health service researcher. FMW and RDN are primary care lung cancer researchers from the UK. MA did the main analysis and engaged in peer debriefing with co-authors as dyads and groups. Co-authors review aspects of the work, such as analysis and coding, theme development, and writing results.

Patient and Public Involvement: The main author is a stage 4 lung cancer patient and a member of a one lung cancer support groups. The research questions were informed by conversations with lung cancer communities. Patient gatekeepers helped recruiting participants by sharing about the study in their support groups. The study will be shared with cancer communities on social media, and specificially in the support group venues.

For peer review only

Results

A total of 40 patients were interviewed. Their mean age was 48 (range 30–75); 12 were male and 28 were female. Interviews were conducted a median of 19.5 months (range 3–152) after diagnosis (Table 1). All participants had a primary diagnosis of metastatic or advanced NSCLC with one driver oncogenic alteration. We noted seven different diagnostic pathways experienced by patients, rather than a single course. These pathways varied primarily by the initial presentation site (primary care, emergency room, etc.) due to the perceived urgency of symptoms (Figure 2).

A. The Experience of Lung Cancer Diagnosis

Emergent themes within the diagnostic intervals (patient, primary care, and secondary care) are detailed below.

1) Patient interva1 (Table 2)

a) Initial concerns about symptoms despite low perception of risk

Prior to diagnosis, lung cancer did not come to mind for most participants, especially as most were younger and non-smokers. Many believed their healthy lifestyle protected them against such illnesses. In contrast, those who smoked suspected lung cancer from the onset of symptoms. The participants recalled experiencing various new symptoms or a change in persisting symptoms that concerned them. Most reported nonspecific symptoms; some were respiratory in nature, while others related to organs and systems due to metastatic spread (e.g., bone pain) or were constitutional (e.g., fatigue, weight loss). Some recalled the symptoms being present up to few months prior to diagnosis. A minority did not recall any symptoms. Diagnosis occurred after imaging for other reasons, such as an injury or trauma.

b) Attribution of symptoms to other causes, and not always seeking care immediately

Participants initially attributed their symptoms to reasons other than lung cancer. Coughing, for example, was explained by forest fire smoke in the air; back pain was attributed to muscle spasm; fatigue was blamed on depression, and shortness of breath with activities on excessive weight. Even hemoptysis raised concern for tuberculosis as a more likely cause. Many participants did not worry initially because the symptoms were perceived as mild or they felt others had similar symptoms, such as dismissing a cough during flu season. Finally, some people did not have health insurance at the time of early symptoms, and the potential cost of health care services deterred them from seeking help.

c) Changes in severity or nature of symptoms prompting care-seeking actions

Participants expressed experiencing a change in their level of concern prompting them to seek medical attention. Reasons included symptoms getting worse, especially after initially improving; not responding to treatments for other suspected illnesses; symptoms lingering; disruptive pain; symptoms developing in combination; alarming symptoms appearing, such as hemoptysis or significant weight loss; and symptoms affecting quality of life or affecting sleep. Sometimes family members or friends had advised the person to seek care after noticing symptoms.

Most individuals initially visited their primary care providers (PCPs) to get help with their symptoms or to determine the reason for the symptoms that had become concerning. Some first visited urgent care, especially when they encountered delays in accessing a PCP. Some patients who had established relationships with specialists consulted with them first: some complained to their ear, nose and throat doctor about their hemoptysis while others complained to their gastroenterologist about their shortness of breath.

2) Primary care interval (Table 3)

a) Doctors initially treated for illnesses other than lung cancer

Participants described that providers were not alarmed by, or sometimes dismissed, their initial symptoms. For many, the initial course of management was the investigation and treatment of benign etiologies. In some cases, initial investigations supported other diagnoses, such as a

For peer review only

respiratory infection from chest X-ray (CXR) or acid reflux confirmed on endoscopy. In other cases, initial tests were normal. Some patients' symptoms were attributed to and treated as other diseases, for example, a shortness of breath was attributed to underlying asthma and treated with inhalers and steroids. Some patients were referred to specialists, such as physical therapy or orthopedics for musculoskeletal complaints. The wait for specialist appointments sometimes took several weeks. Not infrequently, providers used "safety netting", or contingency plans, such as scheduling return visits, follow-up CXR, and trying other treatment plans.

b) Discovery of imaging abnormality, often on CXR and/or chest CT, leading to diagnosis

A major turning point identified by some participants was getting a CXR, either at their request or prompted by their PCP, intended to identify the cause of symptoms. Imaging studies were also ordered when treatment failed or to assess whether previously-noted radiologic findings had been resolved. Occasionally, imaging tests were used to evaluate incidental conditions such as injuries, while other patients received CXR to follow up on nodules seen on previous imaging. Other imaging tests used to evaluate symptoms elsewhere in the body identified lung cancer as an incidental or unexpected finding, such as magnetic resonance imaging (MRI) for back pain or breast-screening MRIs identifying lung lesions.

For many patients, a diagnosis of lung cancer was supported by a chest CT done after an abnormal CXR or to discover the primary site after a metastasis was found. Scheduling the CT scan was often rushed. Sometimes PCPs pushed for this to happen or, when scheduling was delayed, advised patients to go to the emergency room (ER).

c) Severity of symptoms prompting need for emergency care

Some patients went directly to the ER with distressing symptoms such as severe shortness of breath. Others sought care in the ER for symptoms such as headache and back pain as they had no PCP. At times, the patient's condition deteriorated quickly, requiring admission due to hypoxia or losing consciousness with brain tumors causing seizures. Occasionally, delays in diagnostics or the perception that their PCP could not offer much besides office testing prompted the patient to go to ER. Other patients were advised to go to the ER after findings such as a pulmonary embolism or massive brain metastasis. At the ER, it was not uncommon for the patient to be admitted. Some patients demanded urgent consultations from specialists and to be admitted to complete the cancer workup and start treatment.

3) Specialty care interval (Table 4)

a) The pivotal nature of tissue sample collection

Once imaging raised the alarm for cancer, interventional radiologists, pulmonologists, or thoracic surgeons obtained tissue samples. While some patients saw a specialist fairly quickly, others experienced significant delays. Bronchoscopy, needle biopsies, sampling of pleural effusions, and occasionally surgical biopsies were used to clarify if the lesions seen on imaging were cancer, to identify the type of cancer, and to obtain tumor tissue for genetic testing. Results were delivered within a few days. While a bronchoscopy was often uneventful, it sometimes led to major bleeding, collapsed lungs, or the patient requiring resuscitation. Occasionally, concerns over the procedure led to delays in this diagnostic step. When decisions were made to forego

For peer review only - http://bmjopen.bmj.com/site/about/guidelines.xhtml

biopsy, patients felt they were provided false reassurance based on less reliable information, such as the appearance on images and their overall assumed low-risk of cancer.

b) Access to oncologists determined staging but perceived delays led to distress

Patients were referred to an oncologist once diagnosed. The referral was made urgently, often by the PCP or pulmonologist based on imaging findings or following pathology results. It was not uncommon for patients to perceive a delay in making appointments, causing frustration. To identify the right specialist and overcome delays, patients often leveraged personal connections or sought help from family and the cancer community. First meetings with oncologists often involved reviewing the results and setting treatment plans. These were usually short, especially if molecular results were not back. Oncologists often completed the diagnostic workup by ordering additional imaging such as positron emission tomography (PET) scans or brain MRIs. Since our participants had advanced diseases, PET scans often showed metastasis outside the lungs.

c) Genetic testing was crucial in directing patients to targeted treatments

For our participants, molecular testing on tissue or blood samples was obviously an instrumental part of their diagnosis. Realization of a positive mutation was met with relief, as patients were fortunate to be a candidate for targeted therapy. However, molecular testing results sometimes took several weeks or were overlooked by providers. Looking back, some patients described frustration at being given chemotherapy instead of waiting for molecular testing results. Some, however, needed emergency chemotherapy, radiation, or surgery to relieve symptoms.

Discussion

As the first on the subject, this study contributes to the literature on pathways to diagnosis and the intervals of diagnosis among patients with advanced lung cancer on targeted therapy. The participants were mostly young, non-smokers, unlike those in previous research in this area. We used a well-established model to map participants' experiences from their initial realization of symptoms, through contact with health care, and diagnostic workup.[19,20]

Previous studies on this 'patient interval' suggested that atypical or vague symptoms caused delays in knowing when to seek care. Previous research (with participants who were predominantly smokers) noted reluctance among patients to visit their health care provider when symptoms emerge,[6] but this pattern was not reported by the majority of our study participants. Because they were younger than the average age at presentation of lung cancer and/or presented with non-specific symptoms,[4] their concerns were typically attributed initially to benign diseases. Recognizing the symptoms and making a diagnosis can be particularly challenging when a patient has comorbid conditions with symptoms similar to those of lung cancer.[4,23]

Many patients perceived inefficiency and delays in the primary care interval. However, these perceptions were made retrospectively, bringing into question whether an actual delay took place. Some patients felt they had to advocate for themselves to obtain initial diagnostic testing and push for more advanced testing when initial tests were inconclusive. This finding is consistent with the role of self-advocacy in improving the quality of care for patients with cancer.[24,25] Previous studies

For peer review only - http://bmjopen.bmj.com/site/about/guidelines.xhtml

suggested dismissive responses from PCPs may impact patients' decisions to consult care again.[26, 27] In contrast, our participants reported persistence and, at times, sought other providers. Some providers clearly had contingency and follow-up plans, but patients commonly felt they were dismissed without clear "safety netting".[28]

Previous US studies of lung cancer patients have suggested delays occur mainly in the primary care interval through misdiagnosis (and from monitoring nodules) rather than in the specialty-care interval.[29] In contrast, difficulty in accessing secondary care is a major cause for delays in the United Kingdom.[6] Our study found that patients' sense of urgency and perception of unnecessary waiting intensified after receiving imaging diagnosing possible cancer. Many complained about delays in accessing pulmonologists, oncologists, or in results from molecular testing. While these waits were were fairly short and probably had little impact on the overall prognosis, they did appear to intensify patient emotion.

This study has many strengths. It is the first to explore the perspectives of a relatively new group of lung cancer survivors: those on targeted therapies that experience significantly superior outcomes. Interviewees may have been better able to reflect on their diagnostic journey in the absence of side effects from chemo or radiation therapy. Our findings were developed within an existing framework used in research on early diagnosis of cancer by many other countries. Our study also has a few limitations. Only a small proportion of our participants experienced barriers in accessing care due to financial concerns, which may have limited our ability to determine the impact of these factors.[29] Our sampling relied on individuals identified from lung cancer survivor groups, which may have reduced the representativeness, particular of individuals from less affluent backgrounds and over-recruited patients who were more engaged with their disease and diagnostic work up. Also, we did not actively seek to define smoking status during the interviews, thus we omitted characterizing the sample by this factor. Finally, as a qualitative exploration, our study was not equipped to provide insights about frequencies of occurrences, time indicators, or variations between participants based on their characteristics.

Our study has important practical implications. First, lung cancer affects everyone, including those thought to be at low risk. The public must be made aware of this so when new symptoms appear, they will seek healthcare promptly. This advice should be tempered with knowledge of the extremely low probability of cancer in most patients and the poor predictive value of most symptoms. Second, PCPs should be vigilant for rare but serious diseases with similar symptom profiles to benign conditions. "Safety netting" should including sharing diagnostic uncertainty and encouraging patients to return for further assessment when symptoms fail to respond. More precise diagnostic tools would be valuable to PCPs in this difficult task, but ready access to CXR and CT is clearly important. Third, while access to secondary care for serious conditions like cancer may not be a challenge for all patients in the US, the need for coordinating care, communication with patients, and provision of up-to-date standards of practice continue to be an issue. This issue is relevant especially to patients with lung cancer where targeted therapy has changed the disease outcomes in the past few years for patients who have received molecular testing. It's paramount that these new standards of care be available promptly to all patients.

For peer review only

Acknowledgements: The authors would thank LUNGevity, especially Upal Basu Roy, for helping connect with patient advocacy and support groups. They also thank patients and patient advocates Janet Freeman-Daily, Jill Feldman, Ivy Elkins and Tom Carroll, for helping connect us to research participants. They also acknowledge the ROSOneder support group, the ALK-Positive Facebook Support Group and the EGFR Resisters for supporting and promoting this work.

Funding: This research is linked to the CanTest Collaborative, which is funded by Cancer Research UK [C8640/A23385], of which author Fiona Walter is director and authors Richard Neal and Matthew Thompson are associate directors and Co-investigator. Information, conclusions and opinions expressed in this presentation are of the authors and no endorsement is intended or should be inferred.

Competing Interests: The Authors have no conflict of interest to report.

Contribution Statement: MA, MLZS, FMW, RDN, BHLG, MT contributed to the literature review and the conceptualization of the work. MA conducted the interviews. MA conducted the primary analysis of the data. MLZS and MT did peer debriefing and review of analysis with MA individually and in groups. MA, MLZS, FMW, RDN, BHLG, MT all contributed to the writing of the discussion. All the authors reviewed and approved the final version of the study.

Data sharing: Deidentified data will be shared upon request.

For peer review only

References

1. Siegel RL, Miller KD, Jemal A. Cancer statistics, 2017. CA Cancer J Clin. 2017;67(1):7-30.
2. Center for Disease Control and Prevention. Leading Cancer Cases and Deaths, All Races/Ethnicities, Male and Female, 2017. Available at https://gis.cdc.gov/Cancer/USCS/DataViz.html accessed 6/20/2020.
3. Latimer KM, Mott TF. Lung cancer: diagnosis, treatment principles, and screening. *Am Fam Physician*. 2015;91(4):250–256.
4. Dobson C, Russell A, Brown S, Rubin G. The role of social context in symptom appraisal and help-seeking among people with lung or colorectal symptoms: A qualitative interview study. Eur J Cancer Care (Engl). 2018 Feb 8. doi: 10.1111/ecc.12815. [Epub ahead of print] PubMed PMID: 29419943.
5. McLachlan S, Mansell G, Sanders T, Yardley S, van der Windt D, Brindle L, Chew-Graham C, Little P. Symptom perceptions and help-seeking behaviour prior to lung and colorectal cancer diagnoses: a qualitative study. Fam Pract. 2015 Oct;32(5):568-77. doi: 10.1093/fampra/cmv048. Epub 2015 Jun 22. PubMed PMID: 26099812; PubMed Central PMCID: PMC4576759.
6. Neal RD, Robbé IJ, Lewis M, Williamson I, Hanson J. The complexity and difficulty of diagnosing lung cancer: findings from a national primary-care study in Wales. Prim Health Care Res Dev. 2015 Sep;16(5):436-49. doi: 10.1017/S1463423614000516. Epub 2014 Dec 8. PubMed PMID: 25482333.
7. Walter FM, Rubin G, Bankhead C, Morris HC, Hall N, Mills K, Dobson C, Rintoul RC, Hamilton R. Emeryet J. Symptoms and other factors associated with time to diagnosis and stage of lung cancer: a prospective cohort study. *Brit J Cancer*, 2015 Mar 31;112:S6-S13. doi: 10.1038/bjc.2015.30.
8. Jacobsen MM, Silverstein SC, Quinn M, et al. Timeliness of access to lung cancer diagnosis and treatment: A scoping literature review. Lung Cancer. 2017;112:156-164.
9. Goldstraw P, Chansky K, Crowley J, Rami-Porta R, Asamura H, Eberhardt WE, Nicholson AG, Groome P, Mitchell A, Bolejack V. The IASLC Lung Cancer Staging Project: Proposals for revision of the TNM stage groupings in the forthcoming (eighth) edition of the TNM classification for lung cancer. J Thorac Oncol. 2016;11(1):39-51
10. Vinas F, Ben Hassen I, Jabot L, Monnet I, Chouaid C. Delays for diagnosis and treatment of lung cancers: a systematic review. Clin Respir J. 2016;10(3):267-271.
11. Olsson JK, Schultz EM, Gould MK. Timeliness of care in patients with lung cancer: a systematic review. Thorax. 2009;64(9):749-756.
12. Lovly C, Horn L, Pao W. Molecular profiling of lung cancer, My Cancer Genomelast updated March 16, 2018. Available: https://www.mycancergenome.org/content/disease/lung-cancer/
13. Korpanty GJ, Graham DM, Vincent MD, et al. Biomarkers that currently affect clinical practice in lung cancer: EGFR, ALK, Met, ROS-1, and KRAS. Front Oncol 2014;4:204.doi:10.3389/fonc.2014.00204
14. Okamoto I, Morita S, Tashiro N, et al. Real world treatment and outcomes in EGFR mutation-positive non-small cell lung cancer: long-term follow-up of a large patient cohort. Lung Cancer 2018;117:14–19.doi:10.1016/j.lungcan.2018.01.005

For peer review only - http://bmjopen.bmj.com/site/about/guidelines.xhtml

15. Park S, Ahn B-C, Lim SW, et al. Characteristics and outcome of ROS1-positive non-small cell lung cancer patients in routine clinical practice. J Thorac Oncol 2018;13:1373–82.doi:10.1016/j.jtho.2018.05.026.
16. Pacheco JM, Gao D, Smith D, et al. Natural history and factors associated with overall survival in stage IV ALK-rearranged non-small cell lung cancer. J Thorac Oncol 2019;14:691–700.doi:10.1016/j.jtho.2018.12.014.
17. Al Achkar M, Marchand L, Thompson M, Chow LQ, Revere D, Baldwin LM. Unmet needs and opportunities for improving care for patients with advanced lung cancer on targeted therapies: a qualitative study. BMJ open. 2020 Mar 1;10(3):e032639.
18. Carspecken PF. Critical ethnographies from Houston: Distinctive features and directions. Critical ethnography and education. 2001;5:1-26.
19. Walter FM, Scott SE, Webster A, Emery JD. The Andersen Model of Total Patient Delay: a systematic review of its application in cancer diagnosis. J Health Services Research & Policy 2012;17(2):110-118
20. Scott SE, Walter FM, Webster A, Emery JD. The model of pathways to treatment: conceptualization and integration with existing theory. Br J Health Psychol, 2013; 18(1):45-65.
21. Weller D, Vedsted P, Rubin G, Walter F, Emery J, Scott S, Campbell C, Andersen RS, Hamilton W, Olesen F, Rose P, Nafees S, van Rijswijk E, Muth C, Beyer M, Neal RD. The Aarhus Statement: Improving design and reporting of studies on early cancer diagnosis. *BJC* 2012,**106**:1262-1267. DOI 10.1038/bjc.2012.68
22. Coxon, D., Campbell, C., Walter, F.M. *et al.* The Aarhus statement on cancer diagnostic research: turning recommendations into new survey instruments. *BMC Health Serv Res* **18,** 677 (2018). https://doi.org/10.1186/s12913-018-3476-0
23. Carter-Harris L, Hermann CP, Draucker CB. Pathways to a lung cancer diagnosis. J Am Assoc Nurse Pract. 2015 Oct;27(10):576-83. doi: 10.1002/2327-6924.12242. Epub 2015 Mar 12. PubMed PMID: 25772906; PubMed Central PMCID: PMC4567975.
24. Mathews M, Bulman D, Ryan D. The role of patient and physician advocacy in reducing wait times for cancer care: a qualitative analysis. CMAJ open. 2017 Oct;5(4):E773.
25. Jørgensen CR, Thomsen TG, Ross L, et al. What Facilitates "Patient Empowerment" in Cancer Patients During Follow-Up: A Qualitative Systematic Review of the Literature. Qual Health Res 2018;28:292–304.
26. Birt L, Hall N, Emery JD, Banks J, Mills K, Johnson M, Hamilton W, Walter FM. Responding to symptoms suggestive of lung cancer: a qualitative interview study. BMJ Open Respir Res, 2014;1:e000067. doi:10.1136/bmjresp-2014-000067.
27. Walter FM, Birt L, Cavers D, Scott S, Emery J, Burrows N, Cavanagh G, MacKie R, Weller D, Campbell C. 'This isn't what mine looked like': a qualitative study of symptom appraisal and help seeking in people recently diagnosed with melanoma. BMJ Open, 2014;4(7):e005566. doi: 10.1136/bmjopen-2014-005566.
28. Nicholson BD, Mant D, Bankhead C. Can safety-netting improve cancer detection in patients with vague symptoms?. Bmj. 2016 Nov 9;355:i5515.
29. Vidaver RM, Shershneva MB, Hetzel SJ, Holden TR, Campbell TC. Typical time to treatment of patients with lung cancer in a multisite, US-based study. Journal of oncology practice. 2016 Jun;12(6):e643-53.

For peer review only

Table 1. Participant characteristics.

Table 2. Supportive quotes for the patient interval.

Table 3. Supportive quotes for the primary care provider interval.

Table 4. Supportive quotes for the secondary care interval.

Figure 1. The conceptual model of pathway to treatment.

Figure 2. Identified pathways to diagnosis.

Appendix 1. Interview guide.

For peer review only

Table 1. Participant characteristics.

Participant Characterestics	Median (Range) / Count
Age	49 (30-75) years
Gender	
Male	12
Female	28
Race	
White	34
Others (Asian, Hispanic, biracial (Asian and Hispanic))	6
Region in the US	
West	18
Northeast	8
Midwest	7
South	6
Insurance	
Private	34
Medicare	4
Medicaid	2
Time since diagnosis	19.5 (3-152) months
Cancer Stage at Time of Interview	
IV	38
IIIb	2
Mutation	
ALK	20
EGFR	14
Ros1	6

For peer review only

Table 2. Supportive quotes for the patient interval.

**Initial concerns about symptoms despite low perception of risk**
I have not been into a doctor for a medical check-up at all in all that time. I never had any days off taken my entire work experience. (1001)<br>*I looked really healthy and I'm not a smoker. (3005)*<br>*I started seeing symptoms three-four months before diagnosis. I noticed some tightness in my chest. (1003)*<br>*I just had a dry cough that would not go away. (2007)*
**Attribution of symptoms to other causes, and not always seeking care immediately**
There were a lot of forest fires. The air was always really smoky and I thought maybe part of the headaches or not feeling quite right was caused by the smoke. (2013)<br>*I was having some lower back pain in the kidney area and had some other symptoms that made me think maybe I have got kidney stones. (2006)*<br>*Everybody else in the family also seemed to have flu- like illness going on with a cough; cold-cough kind of thing.(1005)*<br>*I was very weak, very lethargic; the worst I ever felt in my life. I tried to self-medicate. I was not insured. (1003)*
**Changes in severity or nature of symptoms prompting care-seeking actions**
Three more weeks went by and the cough continued to get worse to the point where my chest started hurting and I had a little bit of a backache. (1005)<br>*My wife came back from China, she was away for about a month. She said, "Your coughing is different." At the time, I didn't notice anything yet. (2012)*<br>*I coughed a little blood. I am not stupid I knew I had big trouble. There was no question; I called the doctor. (1012)*<br>*I decided, I'm going to go ahead and see my primary care physician to see if maybe she had some more suggestions of what I can do to help this throat situation. (1017)*

For peer review only

Table 3. Supportive quotes for the primary care provider interval.

**Doctors initially treated for illnesses other than lung cancer**
I recall going to see the Primary Care Physician and mentioned, "I'm constantly clearing my throat." They casually dismissed me; the symptom continued. (3002)<br>*I went to the doctor and she did full blood work and said everything looks great. She said the cough was probably just a little bit of a remnant from the cold and typically it can take 3, 4, 5 or even 6 months to go away and not to worry about it too much. (1001)*<br>*I went back to my doctor again and said, "okay, we've tried asthma, we've tried the allergy, here is some reflux medications," which kind of helped. She sent me to my doctor that specializes in reflux. We did an endoscopy. They came back with, "you do have reflux." (3004)*<br>*I kept seeing various doctors and they would always send me home. Like, "Oh, it's a seasonal cold. Oh, it's allergies. Oh, you pulled the muscles from coughing too much, here are some steroids." (1008)*<br>*I went to a walk in clinic two different times and was diagnosed with walking pneumonia. Both of those times, I did have an x-ray of the chest, and it just showed some cloudiness, it didn't show any kind mass. (2007)*<br>*She put me on a different prescription but she said, "If you're not better in a couple of weeks, come back and we'll do a full pulmonary workup and we'll do more diagnostic testing 'cause this was concerning." (3001)*
**Discovery of imaging abnormality, often on CXR or a chest CT led to diagnosis**
The doctor gave me steroids was leaving the room, I said something to the effect of, "I thought I would have to get an X-ray." I'm the one who mentioned the word, "X-ray". (1017)<br>*I went to get an x-ray of my left rib cage. It felt like something was there. I told my doctor that I think I have cancer and I want her to check for cancer. So she obliged. (1009)*<br>*I made an appointment and set me for a chest x-ray. And this is was to me really an important point. There was a radiologist sitting in the booth. He looked at me and from the look on his face I just knew. (1011)*<br>*After the car accident I was taken to a trauma center and they scanned me and said, "You have a broken back and lung cancer." (2009)*<br>*I went back to the doctor the next day and she took a look and she said, "Hmm, I don't like that (swelling in supra-clavicular area)." And she sent me for an ultrasound. (3001)*<br>*I went for a physical to my primary care doctor. He noticed that I had motor deficits in my hands. He suggested that I get an MRI. I actually had to go and see a neurologist in order to get the prescription for an MRI and paid for. (2013)*<br>*As soon as the order went in for the chest x-ray, I went in to have it done. That night my doctor called back and said, "we saw some things on the chest x-ray, we want to get you in for CT scan." So the chest x-ray was a Monday, the CT scan was a Thursday. On the night of the CT scan, she called back and said, "It looks like cancer." (3004)*<br>*She noticed that my breath sounds weren't right. So she ordered a CT and called me the next day and told me that she was going to send me for a PET. She was pretty concerned that it was lung cancer. (1004)*
**Severity of symptoms prompting need for emergency care**
I was scheduled for a CT scan but the next opening wasn't for like 2 or 3 weeks. I was having so much coughing that I couldn't speak or breathe properly. So I called my APNP's office. She advised that I should go to the ER and get a CT scan. (2007)<br>*We scheduled the biopsy for Thursday. Tuesday morning before I could go for the biopsy, I woke up coughing up blood, a considerable amount of blood which was new that it never happened. So I drove myself to the ER*<br>*The second I went in the pulmonologist office, he checked my oxygen and it was 85%. I took his advise and went to the hospital. (1014)*

For peer review only

Table 4. Supportive quotes for the secondary care interval.

**The pivotal nature of tissue sample collection**
The PCP said, "I think you have a problem. You got to go and see a Pulmonologist immediately." Finding a Pulmonologist with an opening is impossible. (3002)<br>*She said it looks like a metastatic disease. She set me up with a biopsy of the lung and a biopsy of the liver. (3003)*<br>*I tried to have a lung biopsy done and I was sitting on the table and the radiologist came in and he said, "I can't biopsy that nodule, no way." The team were all arguing about it over me and finally the radiologist said it is not biopsiable and so I left. They said, well, that probably is not cancer. (1011)*<br>*I had a biopsy of the lungs and ended up with a completely collapsed lung and a chest tube. (1006)*<br>*A senior pulmonologist said, "We suggest drain her lung, drain her effusion and let her out." But the hospitalist was like, "I don't want to let her out until we get a biopsy because she's going to be in the community and it's trying to schedule all of these and she's going to be given and run around and this is an emergent case so I'm leaving her until she can get the biopsy." (1019)*<br>*I had a needle biopsy and he called me a couple of days later, "It's lung cancer and it's adenocarcinoma, and I'm going to send you to an oncologist. (3001)*
**Access to oncologists determined staging but perceived delays led to distress**
I was discharged from the hospital, came home, had a follow-up appointment a few days later with an oncologist who was just part of the healthcare system. They just assigned to me to somebody. (2008)<br>*I was leaving ever more frantic messages and calling again and again and pushing the reception desk. It was about quarter to 12 before I finally convinced her I needed to talk with the doctor today rather than wait, find out how long I might live. (2014)*<br>*I was able to find a lung cancer foundation. And one of the folks there told me about a lung cancer oncologist in an area close to me and said, "You should reach out to them. And tell them I told you to give them a call." And so I did just that. And the doctor called me back. (3002)*<br>*They noted that there were tumors spotted on in my neck region and at that point in time, they wanted to do a full PET scan to figure out what the extent it was. They turned around really quickly. I must say after the original scan, the quickness of my treatment and exploratory work was very fast. (1013)*<br>*I had developed what I had thought was sciatica, but when they did the scan they found out that it was metastasis in my bone that was hitting my sacrum that was kind of causing the sciatic nerve to be inflamed.(2001)*
**Genetic testing was crucial in directing patients to targeted treatments**
I'm grateful my oncologist ordered molecular testing. I know that's becoming standard as care now, it was not quite so much standard as care at that stage. (1001)<br>*So the following week we are supposed to have an appointment but the insurance took a while to approve everything. We postponed that appointment till we got result and the result were ALK positive. (1019)*<br>*When week number 4 went around, (the local oncologist) has not been following up with me. I've been calling you, we still don't have results. I was uncomfortable right around week 6, so I flew and sat down with an oncologist (at a major cancer center) and he basically said well we don't need to wait. Let's do blood test (liquid biopsy). I'll have the results for you in 7 days. (1020)*<br>*I think that somebody dropped the ball at the hospital because the request for the testing wasn't sent until three weeks after they did the surgery they hadn't even requested to do the molecular testing. So when they finally did it still took another few weeks. (1018)*<br>*I had a week of radiation and they were still waiting for the mutation to come back. (3006)*<br>*He wanted me to start chemo treatment immediately because it seemed to be very aggressive whatever this was. Without waiting for the results of any genomic testing and this is still a point of concern for me because, looking back, I feel things work done improperly. We did not wait for the results of the genomic testing. I was started on chemotherapy. (2008)*<br>*The surgery basically gave me a hug and said, there's some really good news, the tumors tested positive for ROS 1, and I had no idea what this means. (3002)*<br>*I'll never forget when my doctor came in and he said, "Hey, you have the ALK mutation." And he said, "You're really lucky." And I'm like, "What do you mean? How am I lucky?" And he was like, "We have this great medicine that was just approved by the FDA. (1008)*

For peer review only

For peer review only

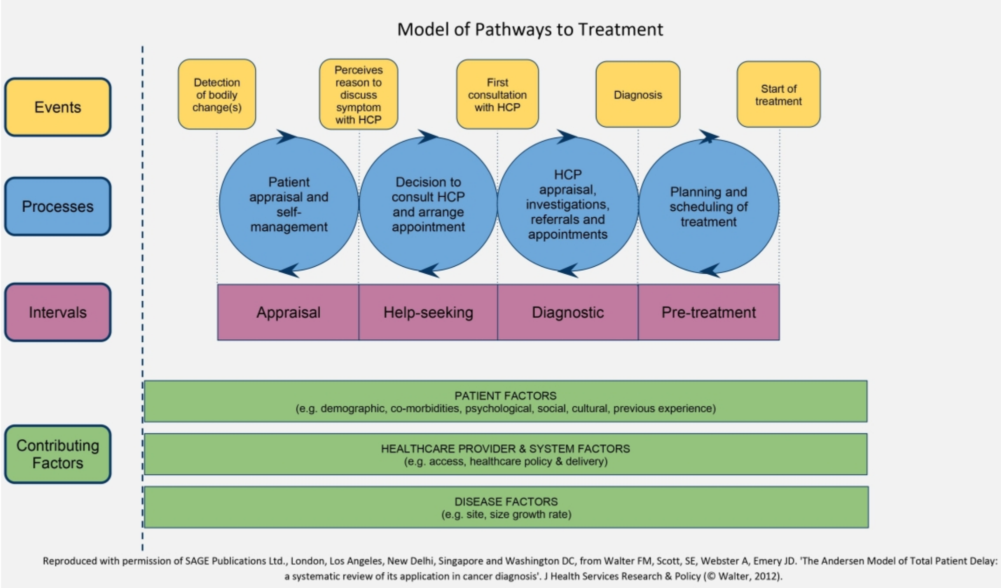

Figure 1. The conceptual model of pathway to treatment.

320x188mm (300 x 300 DPI)

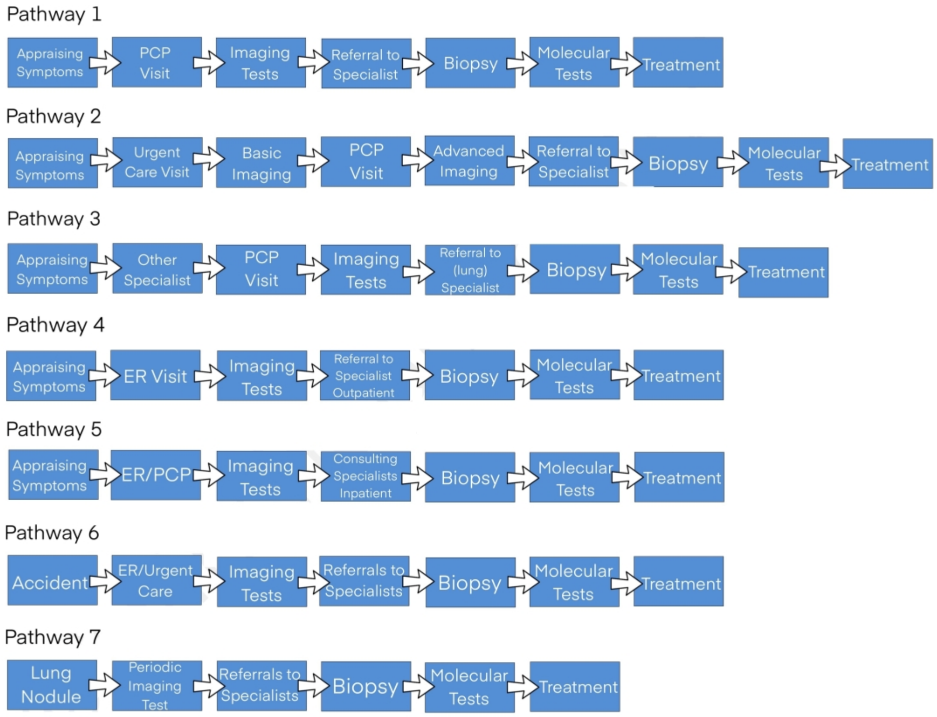

Figure 2. Identified pathways to diagnosis.

670x548mm (132 x 132 DPI)

Intial question.

Share with me the whole story of the cancer diagnosis the way that you would tell it to a friend. Tell me every detail of the whole story of the cancer diagnosis from the very first time when you noticed that something is not right.

Follow up prompts

1. Can you elaborate on some of these specific turning points to reflect on some of the conversations that took place?
2. Besides the [first symptom], what else have you had in the period before the diagnosis of cancer?
3. Tell me more about the conversations with the doctor the first time you had symptoms.
4. Can you share about when your doctor started to be alarmed?
5. Then you had the visit to the [Urgent Care, PCP office] where they did an x-ray, and they found [tumor, fluids, etc.], walk me through the process.
6. Tell me how the results came and how they were conveyed to you. What happened after?
7. They found a tumor. What was its size, and where was it located?
8. How did she share the findings on the [diagnostic test] with you?
9. Please share with me some of your earlier reactions when your primary care doctor gave you the results.
10. How was the process of getting a CT scan?
11. Please walk me through your first interaction with the specialist, the team, or the doctor who was not your primary care.
12. They did a biopsy after that. Please walk me through some of the procedures, the decision around that.
13. Who gave you the cancer diagnosis, and can you walk me through the conversation that took place the first time they confirmed the diagnosis?
14. When the conversation came around the metastatic disease, what were some of your thoughts and feelings at that time?
15. How was your experience with the oncologist?
16. Can you share your conversations with the oncologist?
17. Do you mind sharing what was going on in your mind after the first interaction with the oncologist?
18. When were you told it was [ALK, EGFR, ROS1].
19. Did anybody explain to you what it meant back?
20. Can you explain what [ALK, EGFR, ROS1] means? Assume that I don't know anything about that.
21. Then a few days the results of the [ALK, EGFR, ROS1] came back positive. Tell me how you received the news about [ALK, EGFR, ROS1].
22. Did they offer a management plan, treatment plan early on?
23. How were some of those decisions made to start chemotherapy and radiation?
24. What were conversations around starting the [targeted therapy]?

For peer review only

Standards for Reporting Qualitative Research (SRQR)*

http://www.equator-network.org/reporting-guidelines/srqr/

Title and abstract

	Page/line no(s).
**Title** - Concise description of the nature and topic of the study Identifying the study as qualitative or indicating the approach (e.g., ethnography, grounded theory) or data collection methods (e.g., interview, focus group) is recommended	1
**Abstract** - Summary of key elements of the study using the abstract format of the intended publication; typically includes background, purpose, methods, results, and conclusions	3

Introduction

**Problem formulation** - Description and significance of the problem/phenomenon studied; review of relevant theory and empirical work; problem statement	5
**Purpose or research question** - Purpose of the study and specific objectives or questions	5

Methods

**Qualitative approach and research paradigm** - Qualitative approach (e.g., ethnography, grounded theory, case study, phenomenology, narrative research) and guiding theory if appropriate; identifying the research paradigm (e.g., postpositivist, constructivist/ interpretivist) is also recommended; rationale**	6
**Researcher characteristics and reflexivity** - Researchers' characteristics that may influence the research, including personal attributes, qualifications/experience, relationship with participants, assumptions, and/or presuppositions; potential or actual interaction between researchers' characteristics and the research questions, approach, methods, results, and/or transferability	6
**Context** - Setting/site and salient contextual factors; rationale**	5
**Sampling strategy** - How and why research participants, documents, or events were selected; criteria for deciding when no further sampling was necessary (e.g., sampling saturation); rationale**	5-6
**Ethical issues pertaining to human subjects** - Documentation of approval by an appropriate ethics review board and participant consent, or explanation for lack thereof; other confidentiality and data security issues	5
**Data collection methods** - Types of data collected; details of data collection procedures including (as appropriate) start and stop dates of data collection and analysis, iterative process, triangulation of sources/methods, and modification of procedures in response to evolving study findings; rationale**	5-6

For peer review only - http://bmjopen.bmj.com/site/about/guidelines.xhtml

For peer review only

**Data collection instruments and technologies** - Description of instruments (e.g., interview guides, questionnaires) and devices (e.g., audio recorders) used for data collection; if/how the instrument(s) changed over the course of the study	5-6, appendix
**Units of study** - Number and relevant characteristics of participants, documents, or events included in the study; level of participation (could be reported in results)	6, table 1
**Data processing** - Methods for processing data prior to and during analysis, including transcription, data entry, data management and security, verification of data integrity, data coding, and anonymization/de-identification of excerpts	6
**Data analysis** - Process by which inferences, themes, etc., were identified and developed, including the researchers involved in data analysis; usually references a specific paradigm or approach; rationale**	6
**Techniques to enhance trustworthiness** - Techniques to enhance trustworthiness and credibility of data analysis (e.g., member checking, audit trail, triangulation); rationale**	6

Results/findings

**Synthesis and interpretation** - Main findings (e.g., interpretations, inferences, and themes); might include development of a theory or model, or integration with prior research or theory	6-9
**Links to empirical data** - Evidence (e.g., quotes, field notes, text excerpts, photographs) to substantiate analytic findings	tables 2-4

Discussion

**Integration with prior work, implications, transferability, and contribution(s) to the field** - Short summary of main findings; explanation of how findings and conclusions connect to, support, elaborate on, or challenge conclusions of earlier scholarship; discussion of scope of application/generalizability; identification of unique contribution(s) to scholarship in a discipline or field	9-10
**Limitations** - Trustworthiness and limitations of findings	10

Other

**Conflicts of interest** - Potential sources of influence or perceived influence on study conduct and conclusions; how these were managed	11
**Funding** - Sources of funding and other support; role of funders in data collection, interpretation, and reporting	11

*The authors created the SRQR by searching the literature to identify guidelines, reporting standards, and critical appraisal criteria for qualitative research; reviewing the reference lists of retrieved sources; and contacting experts to gain feedback. The SRQR aims to improve the transparency of all aspects of qualitative research by providing clear standards for reporting qualitative research.

For peer review only - http://bmjopen.bmj.com/site/about/guidelines.xhtml

**The rationale should briefly discuss the justification for choosing that theory, approach, method, or technique rather than other options available, the assumptions and limitations implicit in those choices, and how those choices influence study conclusions and transferability. As appropriate, the rationale for several items might be discussed together.

Reference:

O'Brien BC, Harris IB, Beckman TJ, Reed DA, Cook DA. **Standards for reporting qualitative research: a synthesis of recommendations.** *Academic Medicine*, Vol. 89, No. 9 / Sept 2014 DOI: 10.1097/ACM.0000000000000388

For peer review only

For peer review only - http://bmjopen.bmj.com/site/about/guidelines.xhtml
